# Unraveling Capsule Biosynthesis and Signaling Networks in Cryptococcus neoformans

**DOI:** 10.1128/spectrum.02866-22

**Published:** 2022-10-26

**Authors:** Eun-Ha Jang, Ji-Seok Kim, Seong-Ryong Yu, Yong-Sun Bahn

**Affiliations:** a Department of Biotechnology, College of Life Science and Biotechnology, Yonsei University, Seoul, South Korea; University of Michigan

**Keywords:** Ada2, Bzp4, Gat201, Yap1, transcription factor, kinase

## Abstract

The polysaccharide capsule of Cryptococcus neoformans—an opportunistic basidiomycete pathogen and the major etiological agent of fungal meningoencephalitis—is a key virulence factor that prevents its phagocytosis by host innate immune cells. However, the complex signaling networks for their synthesis and attachment remain elusive. In this study, we systematically analyzed capsule biosynthesis and signaling networks using C. neoformans transcription factor (TF) and kinase mutant libraries under diverse capsule-inducing conditions. We found that deletion of *GAT201*, *YAP1*, *BZP4*, and *ADA2* consistently caused capsule production defects in all tested media, indicating that they are capsule-regulating core TFs. Epistatic and expression analyses showed that Yap1 and Ada2 control Gat201 upstream, whereas Bzp4 and Gat201 independently regulate capsule production. Next, we searched for potential upstream kinases and found that mutants lacking *PKA1*, *BUD32*, *POS5*, *IRE1*, or *CDC2801* showed reduced capsule production under all three capsule induction conditions, whereas mutants lacking *HOG1* and *IRK5* displayed enhanced capsule production. Pka1 and Irk5 controlled the induction of *GAT201* and *BZP4*, respectively, under capsule induction conditions. Finally, we monitored the transcriptome profiles governed by Bzp4, Gat201, and Ada2 under capsule-inducing conditions and demonstrated that these TFs regulate redundant and unique sets of downstream target genes. Bzp4, Ada2, and Gat201 govern capsule formation in C. neoformans by regulating the expression of various capsule biosynthesis genes and chitin/chitosan synthesis genes in a positive and negative manner, respectively. In conclusion, this study provides further insights into the complex regulatory mechanisms of capsule production-related signaling pathways in C. neoformans.

**IMPORTANCE** Over the past decades, human fungal pathogens, including C. neoformans, have emerged as a major public threat since the AIDS pandemic, only to gain more traction in connection to COVID-19. Polysaccharide capsules are rare fungal virulence factors that are critical for protecting C. neoformans from phagocytosis by macrophages. To date, more than 75 proteins involved in capsule synthesis and cell wall attachment have been reported in C. neoformans; however, their complex upstream signaling networks remain elusive. In this study, we demonstrated that Ada2, Yap1, Bzp4, and Gat201 were key capsule-inducing transcriptional regulators. Yap1 and Ada2 function upstream of Gat201, whereas Bzp4 and Gat201 function independently. Genome-wide transcriptome profiling revealed that Bzp4, Gat201, and Ada2 promote capsule production and attachment by positively and negatively regulating genes involved in capsule synthesis and chitin/chitosan synthesis, respectively. Thus, this study provides comprehensive insights into the complex capsule-regulating signaling pathway in C. neoformans.

## INTRODUCTION

Polysaccharide capsules are universal virulence factors for several bacterial and fungal pathogens. For example, Streptococcus pneumoniae, a major etiological agent for community-acquired pneumonia, contains 98 different polysaccharide capsule types which play a pivotal role in its virulence by inhibiting opsonin-mediated phagocytosis (1). In Neisseria meningitidis, which causes bacterial meningitis, the polysaccharide capsule (serogroups A, B, C, W, X, and Y) is the major virulence determinant (2). Mycobacterium tuberculosis, the pathogen which causes tuberculosis, also contains a glycogen-like, α-glucan-type polysaccharide capsule that serves as an antiphagocytic factor (3). The polysaccharide capsule is the major target for the development of vaccines against many bacteria because it is the dominant antigenic surface structure.

Although polysaccharide capsules are virulence determinants commonly found in bacterial pathogens, they are rarely observed in fungal pathogens. Nevertheless, polysaccharide capsules are critical for the survival of Cryptococccus neoformans, a basidiomycete fungal pathogen that causes fatal meningoencephalitis, in both natural environments and susceptible hosts. In the natural environment, the capsule protects the fungus from desiccation and predation by nematodes or amoebae (4). Upon infection, capsule production is further augmented by host-derived signals such as 5% CO_2_, low iron, neutral pH, and tissue culture conditions (5, 6). Within the host, the capsule modulates the immune system in various ways during infection. The capsule not only inhibits inflammatory cytokine production, the antigen-presenting capacity of monocytes, and phagocytosis by macrophages, but also depletes complement components (7, 8). Furthermore, the capsule serves as a reactive oxygen species (ROS) scavenger in macrophages (9). In fact, the mean capsule size and cell wall thickness increase significantly during the course of infection (10) and the deletion of capsule biosynthesis genes significantly attenuates the virulence of C. neoformans (11). Because polysaccharide capsules are uniquely present in C. neoformans among pathogenic yeasts, the observation of encapsulated yeast cells in clinical samples, such as cerebrospinal fluid, is a major indicator of cryptococcosis (12, 13).

Cryptococcal polysaccharide capsules are structurally distinct from other fungal cell wall components and the mammalian extracellular matrix (14). The majority of the cryptococcal capsule (90% to 95%) is composed of glucuronoxylomannan (GXM), which consists of *O-*acetylated α-1,3-linked mannose polymer with xylosyl and glucuronyl side groups (5). Galactoxylomannan (GalXM), composed of α-1,6-linked galactose polymers with mannosyl, xylosyl, and glucuronyl side groups, comprises the remaining 5% to 10% of the capsule (15). GXM and GalXM are synthesized within the cell, transported to the cell wall through vesicles, and noncovalently attached to and polymerized on the cell surface, but they are often shed extracellularly during infection (16). To date, at least 35 genes have been reported to be involved in capsule biosynthesis, including *CAP10*, *CAP59*, *CAP60*, and *CAP64*. All Cap proteins share homology with enzymes involved in polysaccharide biosynthesis and modification (17). *CAP10* encodes a 79-kDa type II membrane protein with an *N*-linked glycosylation site (18). *CAP59* and *CAP60* are involved in the synthesis of GXM (19). *CAP64* and its five homologs have been implicated in GXM xylosylation and acetylation (5). Nevertheless, the specific functions of these Cap proteins in capsule formation remain unclear.

Discovering polysaccharide capsule regulatory signaling pathways is critical for understanding the virulence of C. neoformans. The cAMP/protein kinase A (PKA) and high-osmolarity glycerol response (HOG) pathways have been considered central signal transduction systems to control capsule formation (20, 21). However, recent systematic functional analyses of signature-tagged mutants of 155 transcription factors (TFs), 129 kinases, and 114 phosphatases in C. neoformans have revealed a number of potential signaling components directly or indirectly involved in capsule production (22–24), suggesting that capsule biosynthesis-regulating signaling networks are far more complex than previously expected.

In this study, we systematically analyzed previously identified capsule-regulating signaling TFs and kinases and their related networks under multiple capsule induction conditions, including Dulbecco’s modified Eagle’s (DMEM), Littman’s (LIT), and fetal bovine serum (FBS) media. This study provides further insights into the complex regulatory mechanisms of capsule production-related signaling pathways in C. neoformans.

## RESULTS

### Identification of core capsule regulating transcription factors in *Cryptococcus neoformans*.

**FIG 1:**
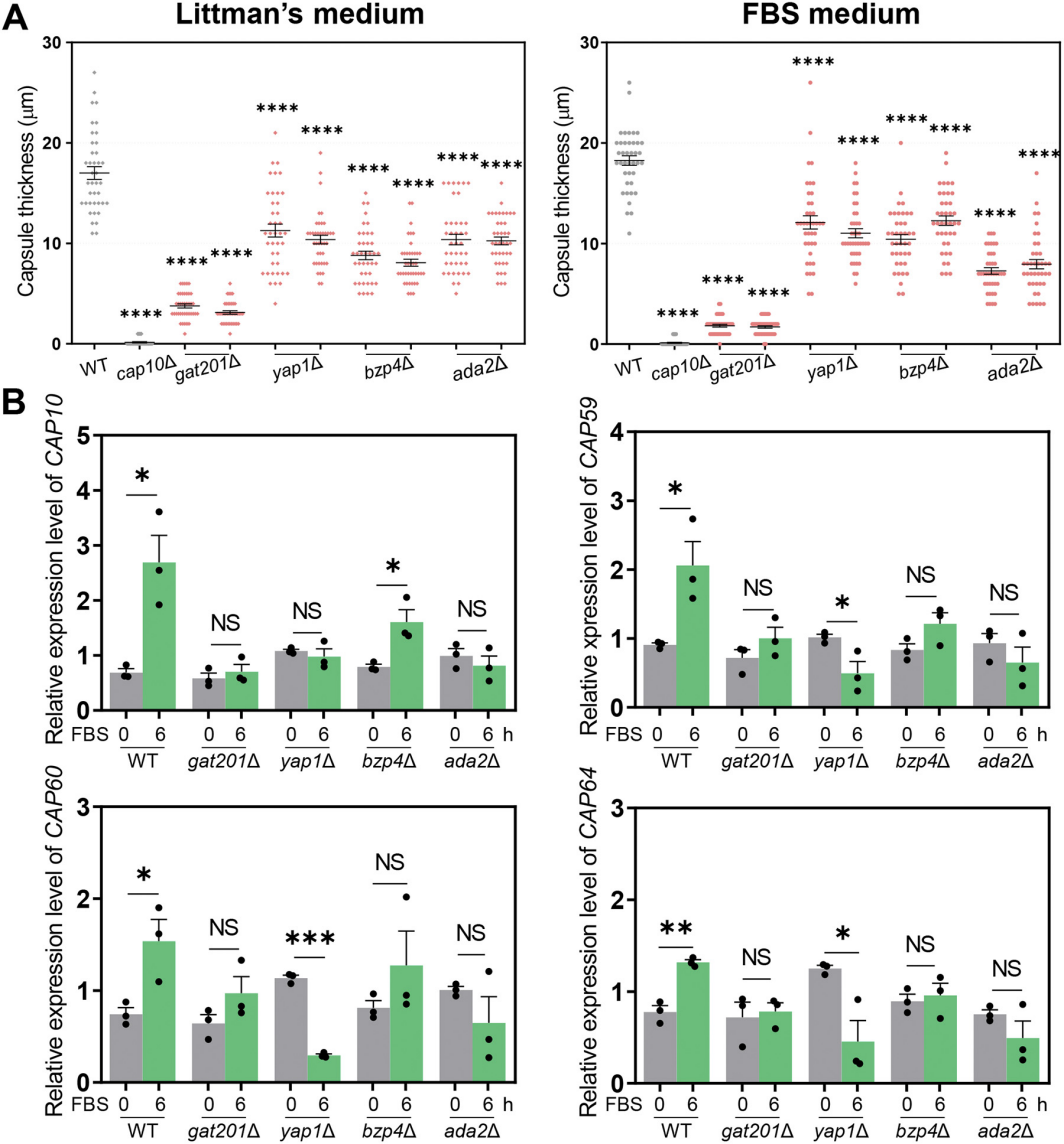
Core transcription factors involved in Cryptococcus neoformans capsule biosynthesis. (A) Wild-type (WT) (H99S), *cap10*Δ (YSB4081), *gat201*Δ (YSB3300, YSB3301), *yap1*Δ (YSB815, YSB1290), *bzp4*Δ (YSB1894, YSB1895), and *ada2*Δ (YSB2381, YSB2382) strains were grown in yeast extract-peptone-dextrose (YPD) liquid medium at 30°C shaking incubator for 16 h, washed with phosphate-buffered saline (PBS), and spotted onto a Littman’s (LIT) solid medium and 10% fetal bovine serum (FBS) solid medium. Cells were incubated for an additional 2 days at 37°C. Each graph indicates relative capsule size of two independent transcription factor mutants which exhibited statistically significant changes in capsule production (±30% difference relative to wild type as the cutoff). Three biologically independent experiments were performed, and representative data are shown here. Each measurement was repeated for 40 cells per condition. Error bars indicate standard deviation. Statistical analysis was performed using one-way analysis of variance (ANOVA) with Bonferroni’s multiple-comparison test. (*, *P* < 0.05; **, *P* < 0.01; ***, *P* < 0.001; ****, *P* < 0.0001). (B) The expression level of capsule-biosynthesis genes was determined using quantitative reverse transcription PCR (qRT-PCR) with cDNA from total RNA samples of WT (H99S), *gat201*Δ (YSB3300), *yap1*Δ (YSB815), *bzp4*Δ (YSB1895), and *ada2*Δ (YSB2382) strains grown in basal YPD medium and 10% FBS medium. *CAP10*, *CAP59*, *CAP60*, and *CAP64* expression levels were normalized by actin gene (*ACT1*) expression. Each strain grown in YPD medium (time zero sample) were resuspended in 10% FBS liquid medium and further incubated for 6 h. Three biological replicate samples with three technical replicates were analyzed using qRT-PCR. Error bars indicate standard deviation. Statistical analysis was performed using Student’s *t* test (*, *P* < 0.05; **, *P* < 0.01; ***, *P* < 0.001; ****, *P* < 0.0001).

We quantitatively measured the capsule size in capsule-inducing media: LIT and 10% FBS for 49 previously reported TF mutants exhibiting altered capsule production levels in solid DMEM at 37°C (22) (Fig. S1 in the supplemental material). The capsule thickness was measured by subtracting the cell diameter from the capsule diameter (total diameter − cell body diameter). We selected TF mutants which showed a ±30% statistically significant difference in capsule thickness compared with the wild-type (WT) strain. Among the 49 TFs, 10 and 3 acted as positive and negative regulators, respectively, in LIT solid medium (Ada2, Bzp4, Gat201, Yap1, Hob3, Fzc1, Fzc30, Fzc46, Usv101, and Nrg1 as positive regulators; Fzc18, Fkh2, and Zfc3 as negative regulators) (Fig. S1). In addition, out of 13 TFs, deletion of four genes (*GAT201*, *YAP1*, *BZP4*, and *ADA2*) led to significantly reduced capsule production in FBS medium (Fig. 1A and Fig. S1). The *gat201*Δ mutant exhibited the most defective capsule production; therefore, we focused on these four TFs for further experiments. However, none of the single mutants exhibited capsule production defects, such as that of the acapsular *cap10*Δ mutant (Fig. 1A), indicating that these TFs may play redundant roles or that multiple other TFs cooperate to control capsule production.

Next, we examined the roles of the four TFs in the regulation of the expression of capsule biosynthesis genes under capsule-inducing conditions (LIT, DMEM, and FBS). We found that the expression of capsule biosynthesis genes, such as *CAP10*, *CAP59*, *CAP60*, and *CAP64*, was generally induced in FBS, LIT, and DMEM media (Fig. S1), further supporting that *CAP* genes are required for capsule formation under all capsule-inducing conditions (11). Notably, the expression levels of *CAP* genes most robustly increased in the 10% FBS medium (Fig. S1). Therefore, we compared *CAP* gene expression using quantitative reverse transcription PCR (qRT-PCR) between the four TF mutants and the wild-type strain in FBS medium (Fig. 1B). The induction of *CAP* gene expression in FBS medium was markedly reduced in the *gat201*Δ, *yap1*Δ, *bzp4*Δ, and *ada2*Δ mutants (Fig. 1B). These results suggest that Gat201, Yap1, Bzp4, and Ada2 cooperate to induce *CAP* gene expression under capsule-inducing conditions.

### Signaling networks of Gat201, Yap1, Bzp4 and Ada2 for capsule production.

**FIG 2:**
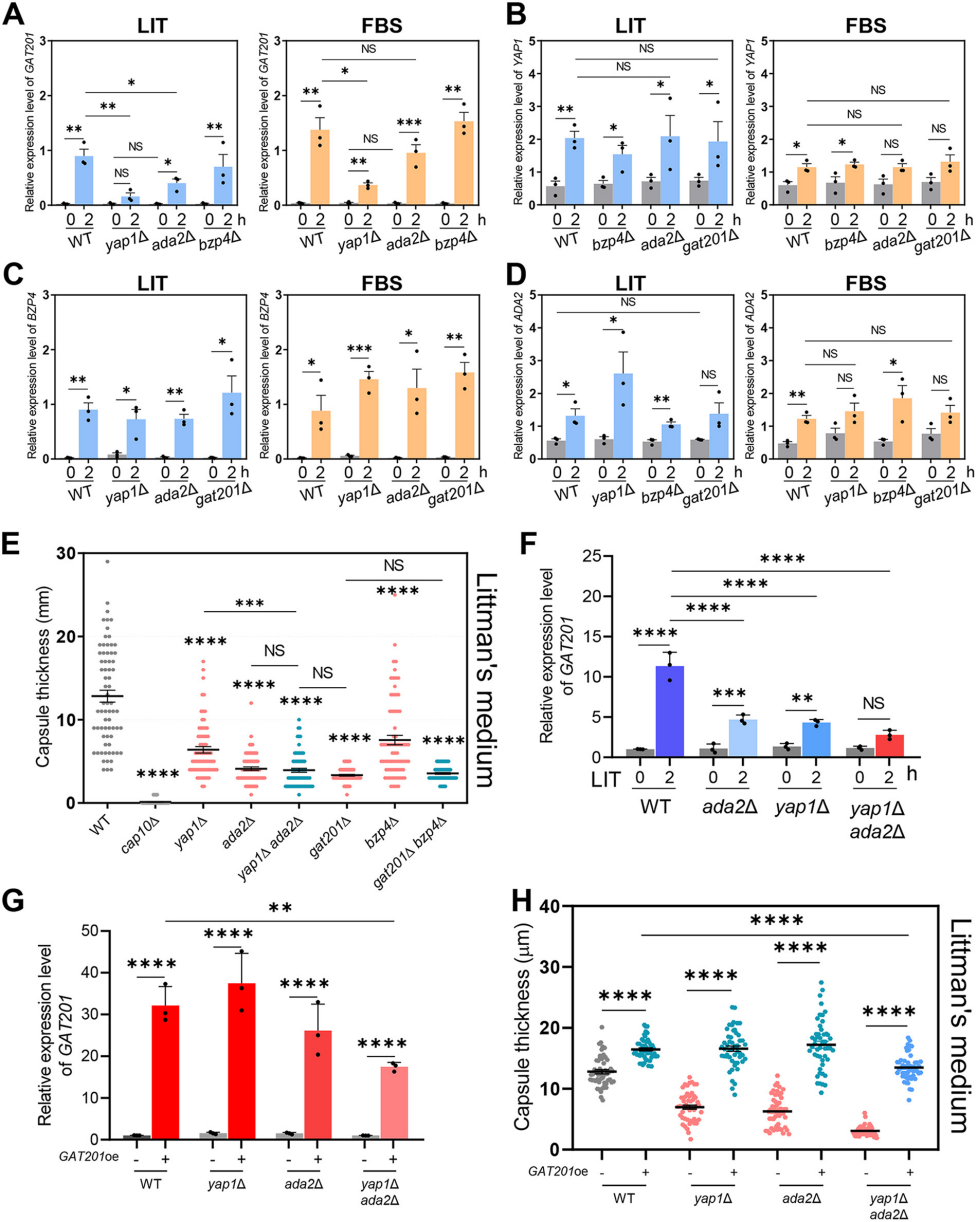
Yap1 and Ada2 coregulate *GAT201* induction under capsule-inducing conditions. The expression levels of *GAT201*, *YAP1*, *ADA2*, and *BZP4* were determined using qRT-PCR with cDNA from total RNA samples of WT (H99S), *gat201*Δ (YSB3300), *yap1*Δ (YSB815), *bzp4*Δ (YSB1895), and *ada2*Δ (YSB2382) strains grown in basal YPD, LIT, and 10% FBS liquid medium. (A) *GAT201*, (B) *YAP1*, (C) *BZP4*, and (D) *ADA2* expression levels normalized to *ACT1* expression. Each strain grown in YPD medium (time zero sample) was resuspended in LIT or FBS liquid medium and further incubated for 2 h. Three biological replicate samples with three technical replicates were analyzed using qRT-PCR. Error bars indicate standard deviation. Statistical analysis was performed using Student’s *t* test. (E) WT (H99S), *cap10*Δ (YSB4081), *yap1*Δ (YSB815), *ada2*Δ (YSB2382), *yap1*Δ *ada2*Δ (YSB6054), *gat201*Δ (YSB3300), *bzp4*Δ (YSB1895), and *gat201*Δ *bzp4*Δ (YSB6052) strains were grown in YPD liquid medium at 30°C for 16 h, washed with PBS, and spotted onto LIT solid medium. The cells were further incubated for 2 days at 37°C. Three biologically independent experiments were performed, and representative data are shown. Each measurement was repeated for 70 cells for each condition. Error bars indicate standard deviation. Statistical analysis was performed using one-way ANOVA with Bonferroni’s multiple-comparison test. (F) *GAT201* expression levels were determined using qRT-PCR with cDNA from total RNA samples of WT (H99S), *ada2*Δ (YSB2382), *yap1*Δ (YSB815), and *yap1*Δ *ada2*Δ (YSB6054) strains grown in basal YPD and LIT medium. *GAT201* expression levels were normalized to *ACT1* expression. Each strain grown in YPD medium (time zero sample) was resuspended in LIT liquid medium and further incubated for 2 h. Three biological replicate samples with three technical replicates were analyzed using qRT-PCR. Error bars indicate standard deviation. Statistical analysis was performed using one-way ANOVA with Bonferroni’s multiple-comparison test. (G) *GAT201* overexpression by the *H3* promoter replacement was confirmed by qRT-PCR. Each strain was cultured in YPD liquid medium at 30°C for 16 h and subcultured in fresh YPD liquid medium until the OD_600_ reached 0.6 to 0.8. *GAT201* expression levels were normalized to *ACT1* expression. Total RNA extracted from three biological replicate samples was analyzed using qRT-PCR with three technical replicates. Mean values are shown, error bars indicate standard deviation. Statistical analyses were performed using Student’s *t* test. (H) *GAT201* overexpression strains generated in wild-type, *ada2*Δ, *yap1*Δ, or *yap1*Δ *ada2*Δ strain backgrounds were grown in YPD liquid medium at 30°C for 16 h, washed with PBS, and spotted onto LIT solid medium. The cells were further incubated for 2 days at 37°C. Each measurement was repeated on 50 cells for each condition. Mean values are shown with error bars indicating standard deviation. Statistical analyses were performed using Student’s *t* test. *, *P* < 0.05; **, *P* < 0.01; ***, *P* < 0.001; ****, *P* < 0.0001 for all panels.

To identify potential epistatic relationships between *GAT201*, *YAP1*, *BZP4*, and *ADA2* for capsule production, the expression levels of the four TF genes were measured in each deletion mutant in LIT and FBS media. *GAT201* was strongly induced in both LIT and FBS media in the wild-type strain, but its induction significantly reduced in *yap1*Δ and *ada2*Δ mutants (Fig. 2A). *YAP1* deletion more strongly reduced *GAT201* induction than *ADA2* deletion, suggesting that Yap1 and Ada2 play major and minor roles, respectively, in inducing *GAT201* expression under capsule-induction conditions. In contrast, Bzp4 was dispensable for *GAT201* induction in both the LIT and FBS media (Fig. 2A). Notably, *YAP1*, *BZP4*, and *ADA2* were also induced by both LIT and FBS media in the wild-type strain, but unlike *GAT201*, their induction was not markedly affected by mutations in the other three TFs (Fig. 2B to D). Collectively, these results indicate that *GAT201* induction in response to capsule-inducing conditions was regulated by Yap1 and Ada2, but *BZP4*, *YAP1*, and *ADA2* were independently regulated.

To further elucidate the epistatic relationships between Gat201, Yap1, Bzp4, and Ada2, the following double mutant strains were constructed: *yap1*Δ *ada2*Δ, *gat201*Δ *bzp4*Δ, *ada2*Δ *bzp4*Δ, *gat201*Δ *ada2*Δ, and *yap1*Δ *bzp4*Δ. We examined capsule production levels in these double mutants in LIT and FBS media. In FBS medium, the *yap1*Δ *ada2*Δ mutants showed reduced capsule production compared with each single mutant (Fig. 2E and Fig. S2). Notably, the capsule production level of the *yap1*Δ *ada2*Δ mutant was almost equivalent to that of the *gat201*Δ mutant (Fig. 2E), further suggesting that Yap1 and Ada2 cooperate upstream of Gat201 for capsule induction. Supporting this finding, *GAT201* induction in LIT and FBS media was more strongly reduced in *yap1*Δ *ada2*Δ mutants than in each single mutant (Fig. 2F and Fig. S2). Based on the results showing that the induction of *BZP4* and *GAT201* was mutually exclusive (Fig. 2A and C), we hypothesized that *BZP4* deletion would further reduce the capsule production level of the *gat201*Δ mutant. However, capsule production in the *gat201*Δ *bzp4*Δ mutant was almost equivalent to that in the *gat201*Δ mutant (Fig. 2E and Fig. S2). These results suggest that Bzp4 is regulated independently from Gat201 but may serve as a co-TF to support the full function of Gat201 in capsule formation.

To further confirm whether Gat201 works downstream of Yap1 and Ada2, we tested whether *GAT201* overexpression (*GAT201*^oe^) can rescue capsule production defects in *ada2*Δ, *yap1*Δ, and *yap1*Δ *ada2*Δ mutant strains. To this end, we generated histone 3 (H3) promoter-driven *GAT201* overexpression (*GAT201*^oe^) strains in the wild-type, *ada2*Δ, *yap1*Δ, and *yap1*Δ *ada2*Δ strain backgrounds (Fig. S2). The constitutively active H3 promoter was able to overexpress *GAT201* by approximately 26- to 37-fold in the wild-type, *ada2*Δ, and *yap1*Δ strains (Fig. 2G). However, *GAT201* overexpression levels were lower in the *yap1*Δ *ada2*Δ double mutants compared to those in the wild-type and *ada2*Δ and *yap1*Δ single-mutant strains (Fig. 2G), suggesting that Ada2 and Yap1 regulate *GAT201* expression by affecting its mRNA stability. Strikingly, we found that *GAT201*^oe^ dramatically enhanced capsule production in the wild-type strain and completely rescued capsule defects in the *ada2*Δ, *yap1*Δ, and *ada2*Δ *yap1*Δ mutants in both LIT and FBS media (Fig. 2H and Fig. S2). In fact, the capsule levels observed in the *GAT201*^oe^ *ada2*Δ and *GAT201*^oe^ *yap1*Δ strains were almost equivalent to those observed in the *GAT201*^oe^ strain. These results further corroborate the hypothesis that Gat201 functions downstream of Yap1 and Ada2 to regulate capsule production.

**FIG 3:**
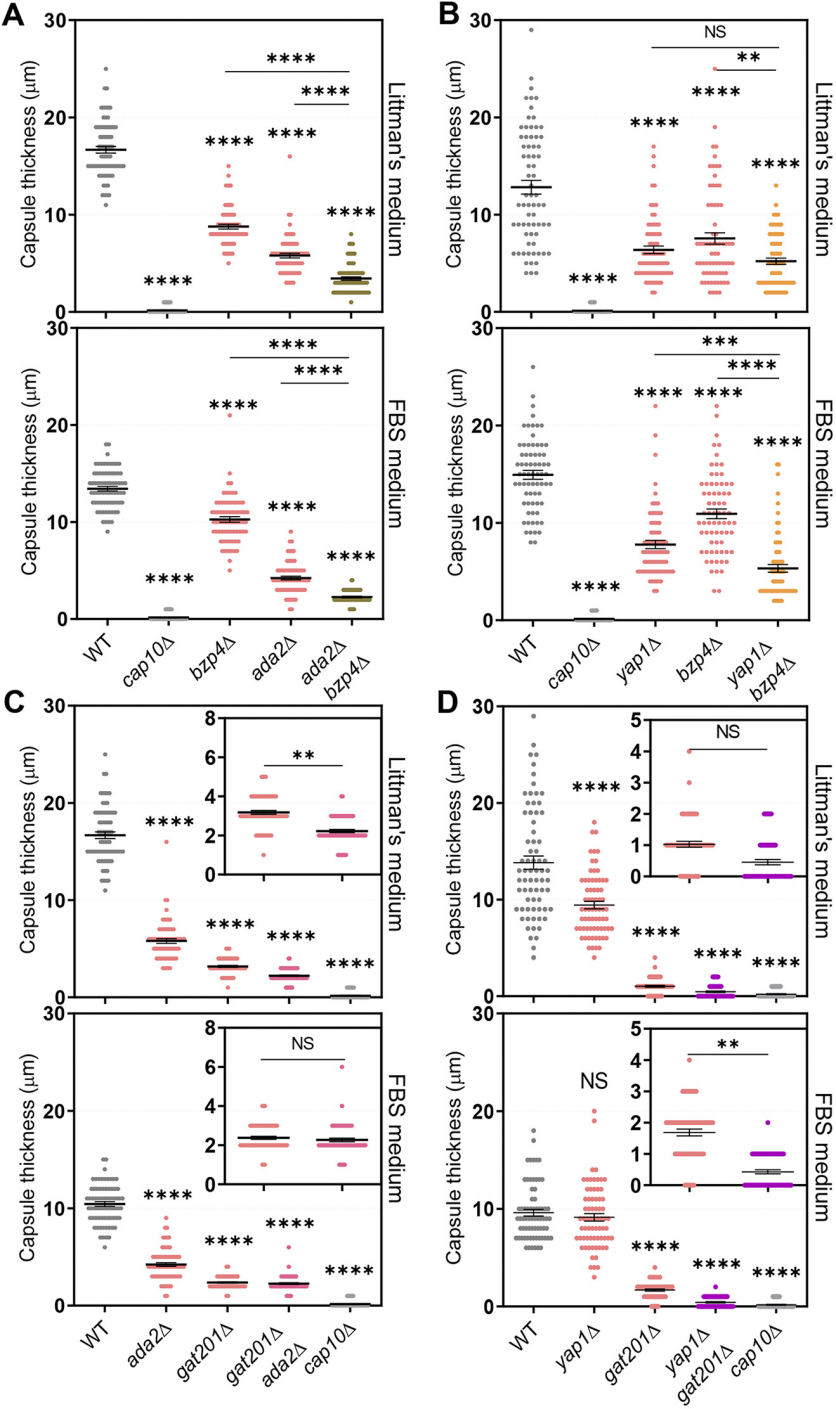
*BZP4* regulates capsule production independently of Ada2 and Yap1. The following strains were grown in YPD liquid medium at 30°C for 16 h, washed with PBS, and spotted onto a Littman’s (LIT) solid medium and 10% fetal bovine serum (FBS) solid medium: (A) WT (H99S), *cap10*Δ (YSB4081), *bzp4*Δ (YSB1895), *ada2*Δ (YSB2382), and *ada2*Δ *bzp4*Δ (YSB6169), (B) WT (H99S), *cap10*Δ (YSB4081), *yap1*Δ (YSB815), *bzp4*Δ (YSB1895), and *yap1*Δ *bzp4*Δ (YSB6055), (C) WT (H99S), *ada2*Δ (YSB2382), *gat201*Δ (YSB3300), *gat201*Δ *ada2*Δ (YSB6167), and *cap10*Δ (YSB4081) (D) WT (H99S), *yap1*Δ (YSB815), *gat201*Δ (YSB3300), *yap1*Δ *gat201*Δ (YSB6695), and *cap10*Δ (YSB4081). (C and D) Small graphs indicate *gat201*Δ and double mutants. Cells were further incubated for 2 days at 37°C. Three biologically independent experiments were performed, and representative data are shown here. Each measurement was repeated for 70 cells per condition. Error bars indicate standard deviation. Statistical analysis was performed using one-way ANOVA with Bonferroni’s multiple-comparison test. *, *P* < 0.05; **, *P* < 0.01; ***, *P* < 0.001; ****, *P* < 0.0001.

Supporting the hypothesis that Yap1 and Ada2 cooperate to regulate Gat201, the *ada2*Δ *bzp4*Δ and *yap1*Δ *bzp4*Δ double mutants showed greater reductions in capsule production than each single mutant (Fig. 3A and 3B), consistent with the result that *BZP4* induction was not regulated by Ada2 and Yap1 under capsule-inducing conditions (Fig. 2C). Notably, the *gat201*Δ *ada2*Δ mutants showed slightly more reduced capsule production in the LIT medium than the *gat201*Δ mutant (Fig. 3C), indicating that Ada2 may have some minor Gat201-independent roles in capsule production. Similarly, the *yap1*Δ *gat201*Δ mutants showed slightly more reduced capsule production in FBS medium than the *gat201*Δ mutant (Fig. 3D), indicating that Yap1 may have some Gat201-independent roles in capsule production.

### Cellular localization of Bzp4, Yap1, Ada2, and Gat201 during capsule production.

**FIG 4:**
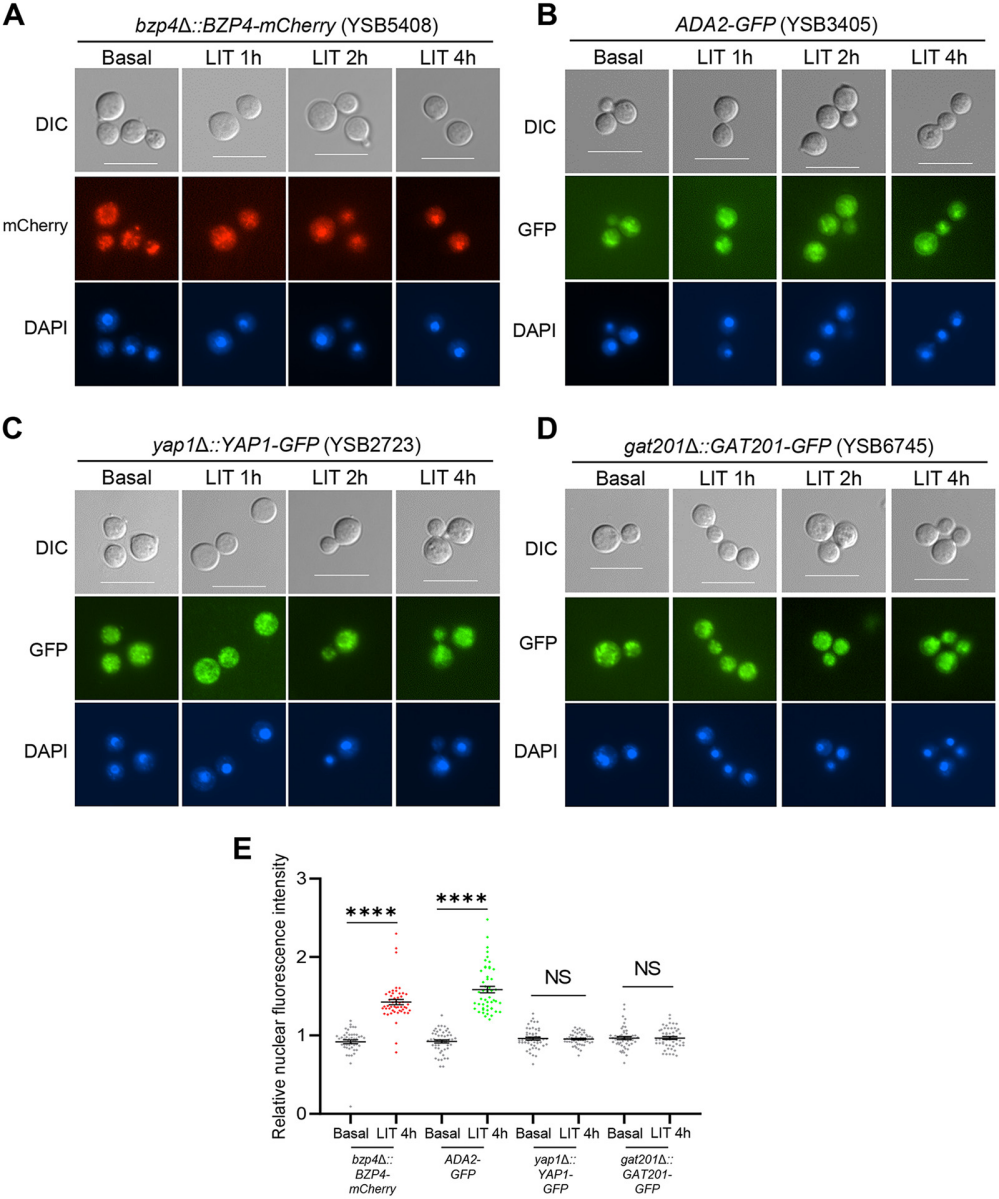
Bzp4 and Ada2 are localized in nucleus under capsule-inducing conditions. (A) *bzp4*Δ::*BZP4-mCherry* (YSB5408), (B) *ADA2-GFP* (YSB3405), (C) *yap1*Δ*::YAP1-GFP* (YSB2723), and (D) *gat201*Δ*::GAT201-GFP* (YSB6745) were cultured in YPD liquid medium at 30°C for 16 h and subcultured in fresh YPD liquid medium until OD_600_ reached 0.6 to 0.8. Subcultured cells were washed with PBS and resuspended in 40 mL LIT medium. After suspension, cells were incubated at 37°C in a shaking incubator and a portion of them (1 mL) was sampled at each time point (1, 2, and 4 h) and fixed in paraformaldehyde. Fixed cells were stained with Hoechst for nuclear staining and observed through differential interference contrast (DIC) microscopy (scale bar = 10 μm). RFP and GFP indicate red and green fluorescent protein, respectively. (E) Quantification of nuclear-localized fluorescent proteins. Relative nuclear fluorescence intensity was calculated as a ratio of the average fluorescence intensity of the nucleus to the average fluorescence intensity of the entire cell. Each measurement was repeated on 50 cells for each condition. Mean values are shown, error bars indicate standard deviation. Statistical analyses were performed using Student’s *t* test. *, *P* < 0.05; **, *P* < 0.01; ***, *P* < 0.001; ****, *P* < 0.0001.

We recently reported that Bzp4 also serves as a major TF in melanin production (25). In response to nutrient starvation—a key melanin-inducing signaling pathway—Bzp4 is translocated from the cytoplasm into the nucleus, governing a plethora of downstream effector genes, including laccase (*LAC1*) (25). Therefore, we examined whether Bzp4 also localized in the nucleus in response to capsule-inducing signals. The *bzp4*Δ::*BZP4*-*mCherry* strain with the mCherry protein fused in-frame to the C terminus of Bzp4 (25) partially restored capsule production compared with the *bzp4*Δ mutant (Fig. S3), indicating that Bzp4-mCherry was partially functional for capsule production. The Bzp4-mCherry protein was distributed throughout the cell under basal conditions but was translocated to the nucleus within 2 h of transition to LIT medium (Fig. 4A).

We also constructed *gat201*Δ::*GAT201*-*GFP* and *yap1*Δ::*YAP1*-*GFP*, where the green fluorescent protein (GFP) was fused in-frame to the C terminus of Gat201 or Yap1, and *ADA2-GFP* with GFP tagged to the C terminus of Ada2. All these strains exhibited normal capsule production (Fig. S3), suggesting that the fluorescently tagged proteins were functional. Like Bzp4, Ada2-GFP was distributed in both the cytoplasm and nucleus under basal conditions but was translocated to the nucleus within 4 h in response to the capsule-inducing signal (Fig. 4B). In contrast, Gat201 and Yap1 localized in both the cytoplasm and nucleus under basal or capsule-inducing conditions (Fig. 4C and D). To quantify GFP/mCherry expression, we measured the average fluorescence intensity of the whole cell and the nucleus and calculated its ratio. Supporting the qualitative data shown in Fig. 4A to D, we found that the relative nuclear fluorescence intensity of Bzp4-mCherry and Ada2-GFP significantly increased from ~0.9 to ~1.5 under capsule-inducing conditions (LIT medium). In contrast, the relative nuclear fluorescence intensities of Yap1-GFP and Gat201-GFP under basal conditions (YPD medium) were similar to those under capsule-inducing conditions (Fig. 4E). These results indicate that Bzp4 and Ada2, but not Gat201 and Yap1, translocate from the cytoplasm to the nucleus during capsule production.

### Upstream regulators of Gat201, Yap1, Bzp4, and Ada2 for capsule production.

**FIG 5:**
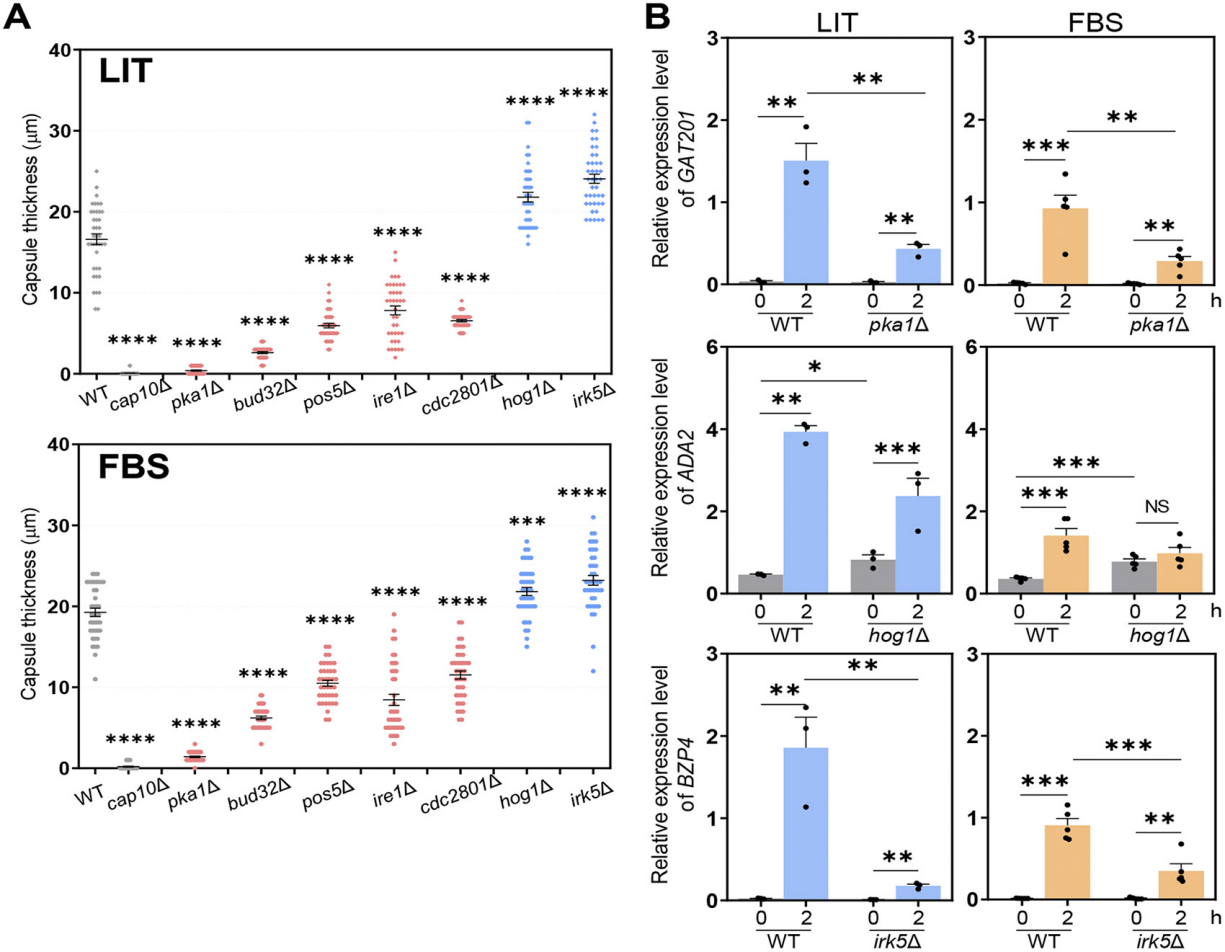
Core kinases involved in C. neoformans capsule biosynthesis. (A) WT (H99S), *cap10*Δ (YSB4081), *pka1*Δ (YSB188), *bud32*Δ (YSB1969), *pos5*Δ (YSB3714), *ire1*Δ (YSB552), *cdc2801*Δ (YSB3699), *hog1*Δ (YSB64), and *irk5*Δ (YSB2952) strains were grown in YPD liquid medium at 30°C in shaking incubator for 16 h, washed with PBS, and spotted onto LIT solid medium and 10% FBS solid medium. Cells were further incubated for 2 days at 37°C. Each graph indicates relative capsule size of transcription factor mutants which exhibited statistically significant changes in capsule production (±30% difference relative to the wild type as cutoff). Three biologically independent experiments were performed, and representative data are shown here. Each measurement was repeated for 40 cells per condition. Error bars indicate standard deviation. Statistical analysis was performed using one-way ANOVA with Bonferroni’s multiple-comparison test. Expression levels of (B) *GAT201*, *ADA2*, and *BZP4* were determined using qRT-PCR with cDNA from total RNA samples of WT (H99S), *pka1*Δ (YSB188), *hog1*Δ (YSB64), and *irk5*Δ (YSB2952) strains grown in basal YPD and LIT. *GAT201*, *ADA2*, and *BZP4* expression levels were normalized by *ACT1* expression. Each strain grown in YPD medium (time zero sample) was resuspended in LIT liquid medium and further incubated for 2 h. Three biological replicate samples with three technical replicates were analyzed using qRT-PCR. Error bars indicate standard deviation. Statistical analysis was performed using Student’s *t* test. *, *P* < 0.05; **, *P* < 0.01; ***, *P < *0.001; ****, *P < *0.0001 for all panels.

Next, we focused on the upstream signaling components which regulate the core TFs involved in capsule induction in C. neoformans. In our previous study, we found that 52 kinases were involved in capsule production in DMEM (22). To identify potential candidate kinases for the regulation of the four capsule-regulating TFs, we first measured the capsule production levels of the 52 kinase mutants in LIT and FBS media. Among these, seven kinase mutants (*pka1*Δ, *bud32*Δ, *pos5*Δ, *ire1*Δ, *cdc2801*Δ, *hog1*Δ, and *irk5*Δ) showed consistently altered capsule production levels in both LIT and FBS media (Pka1, Bud32, Pos5, Ire1, and Cdc2801 as positive regulators; Hog1 and Irk5 as negative regulators) (Fig. 5A). Therefore, we hypothesized that these seven kinases are potential upstream regulators of Gat201, Yap1, Bzp4, and Ada2.

Next, we measured the levels of *GAT201*, *YAP1*, *ADA2*, and *BZP4* in kinase mutants under capsule-inducing conditions (Fig. 5B and Fig. S4). *GAT201* induction was significantly reduced in the *pka1*Δ mutant in both LIT and FBS media (Fig. 5B). However, *YAP1* and *ADA2* induction remained largely unaltered in the *pka1*Δ mutant in LIT or FBS media (Fig. S4). We found that basal expression levels of *ADA2* increased in the *hog1*Δ mutant (Fig. 5B), consistent with a previous report showing that Hog1 mitogen-activated protein kinase (MAPK) negatively regulates Ada2 (22). In contrast, *BZP4* expression was markedly reduced in the *irk5*Δ mutant under capsule-inducing conditions (Fig. 5B). This was unexpected because the *irk5*Δ mutant produced more capsules than the wild-type strain (Fig. 5A). These results indicated that Irk5 may play both positive and negative roles in capsule production in C. neoformans.

### Downstream networks of Gat201, Yap1, Bzp4, and Ada2 for capsule production.

Next, we aimed to elucidate the downstream signaling network governed by capsule-inducing core TFs. We performed RNA sequencing (RNA-seq)-based transcriptome analysis of wild-type and *bzp4*Δ, *ada2*Δ, and *gat201*Δ mutant strains under both basal and capsule-inducing conditions. We did not include the *yap1*Δ mutant in this analysis because Yap1 appeared to mainly act upstream of Gat201, and Yap1-dependent transcriptome profiles were partly reported (26). We also decided to use LIT medium rather than FBS for the capsule-inducing condition because LIT is a better defined medium than FBS. Because *BZP4*, *ADA2*, and *GAT201* expression was highly induced after 2 h of incubation in LIT medium, we monitored the transcriptome profiles of wild-type, *bzp4Δ*, *ada2*Δ, and *gat201*Δ mutant cells grown to the mid-log phase in YPD medium (basal condition) and cells were subsequently incubated for 2 h in LIT medium. When wild-type cells were incubated for 2 h in LIT medium, we found that 1,631 genes were differentially regulated (cutoff: 2-fold change) at statistically significant levels (*P* < 0.05) (Fig. S5). The expression levels of genes involved in integral membrane components, transmembrane transporters, and carbohydrate metabolic processes significantly increased (Fig. S5), implying that polysaccharide capsule production and its transportation outside the cell actively occur under capsule-inducing conditions.

**FIG 6:**
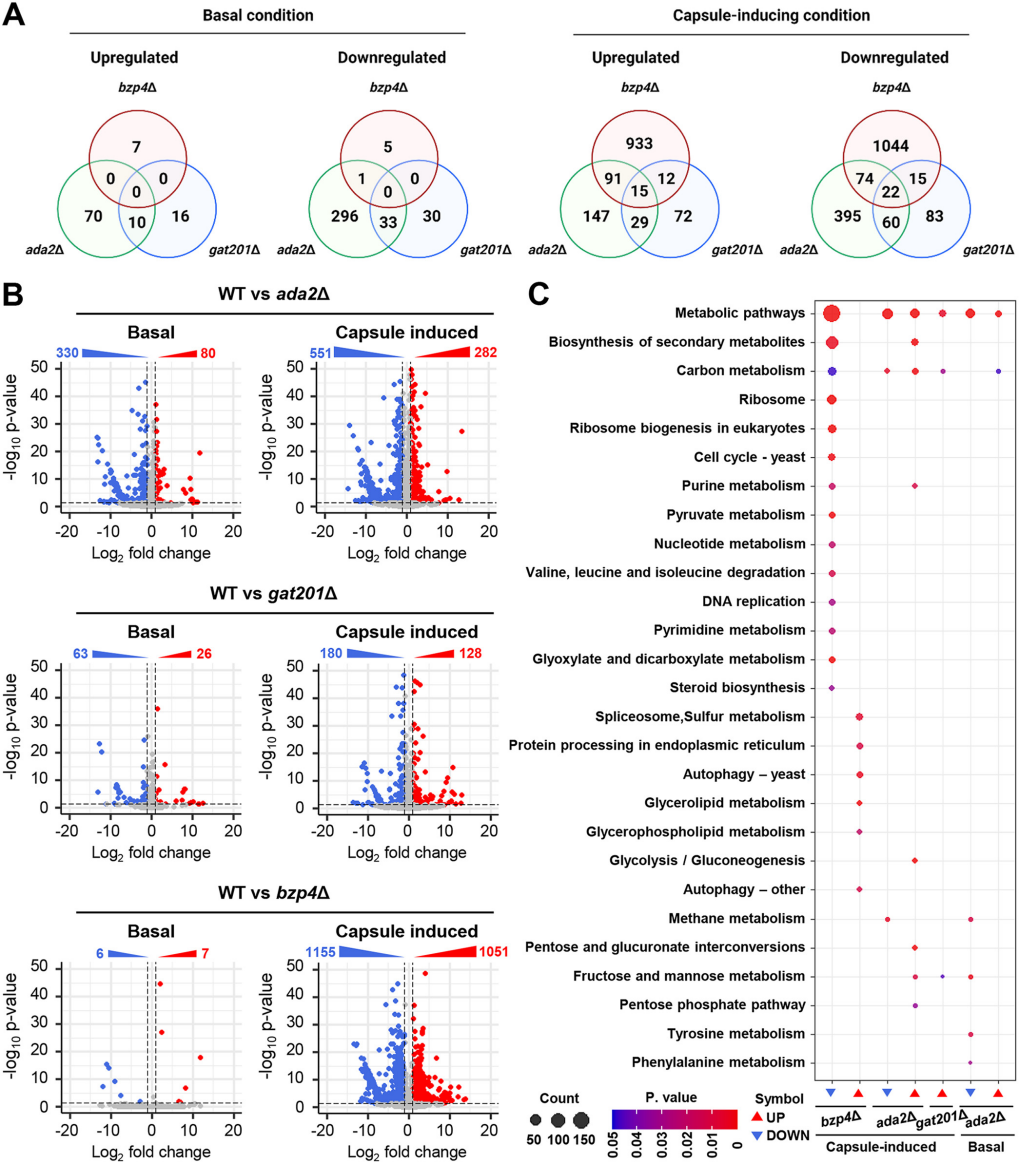
Capsule-inducing condition affects gene expression patterns of wild-type strain, *bzp4*Δ, *ada2*Δ, and *gat201*Δ mutants. (A) Transcriptome profiles governed by Bzp4, Ada2, and Gat201 under basal (YPD) and capsule-inducing (LIT) conditions. The numbers of genes whose expression was significantly up- or downregulated at least 2-fold in the *bzp4*Δ, *ada2*Δ, and *gat201*Δ mutants compared with the WT strain under basal (YPD) or capsule-inducing conditions (LIT) are indicated in Venn diagrams. (B) The cutoff ranges of the fold change were 2 with a *P* value of <0.05 calculated by each set. A volcano plot of the RNA data was created using DESeq2 and plotted using R package. (C) Significant differences in KEGG pathway analysis are shown between basal and capsule-inducing conditions. Mutants which have no significant pathway are not shown.

Principal-component analysis (PCA) and hierarchical clustering of the heatmap clearly showed that a group of genes regulated by Gat201 and Ada2 under capsule-inducing conditions is highly correlated, but distinct from those regulated by Bzp4 (Fig. S6), further confirming the regulatory connection between Gat201 and Ada2. As previously reported (25), deletion of *BZP4* did not significantly alter gene expression under basal conditions (seven upregulated and six downregulated genes; 2-fold change cutoff, *P* < 0.05). Similarly, deletion of *GAT201* led to only a minor change in gene expression (26 upregulated and 63 downregulated genes) under basal conditions. However, *ADA2* deletion resulted in marked changes in transcriptome profiles, even under basal conditions (80 upregulated and 330 downregulated genes) (Fig. 6A and B). This indicates that Bzp4 and Gat201 are not major transcriptional regulators required for growth under basal conditions. However, under capsule-inducing conditions, deletion of *BZP4* led to a dramatic change in transcriptome profiles (1,051 upregulated and 1,155 downregulated genes) (Fig. 6B), reflecting that Bzp4 is translocated from the cytoplasm to the nucleus under capsule-inducing conditions. In contrast, deletion of *GAT201* controlled a smaller number of genes (128 upregulated and 180 downregulated genes) under capsule-inducing conditions. Deletion of *ADA2* regulated an intermediate number of genes (282 upregulated and 551 downregulated genes) under capsule-inducing conditions (Fig. 6B). Through KEGG pathway analysis, we found that Bzp4 is involved in more diverse metabolic pathways than Ada2 or Gat201 under capsule-inducing conditions (Fig. 6C). In particular, the expression of pathway genes associated with the biosynthesis of secondary metabolites decreased significantly, which is expected to be associated with the decrease in polysaccharide synthesis involved in capsule formation (Fig. 6C). In addition, the expression of genes involved in carbon metabolism decreased in *bzp4*Δ, *ada2*Δ, and *gat201*Δ mutant strains under capsule-induction conditions, which is expected to reduce the synthesis of polysaccharides required for capsule formation, resulting in decreased capsule formation (Fig. 6C).

**FIG 7:**
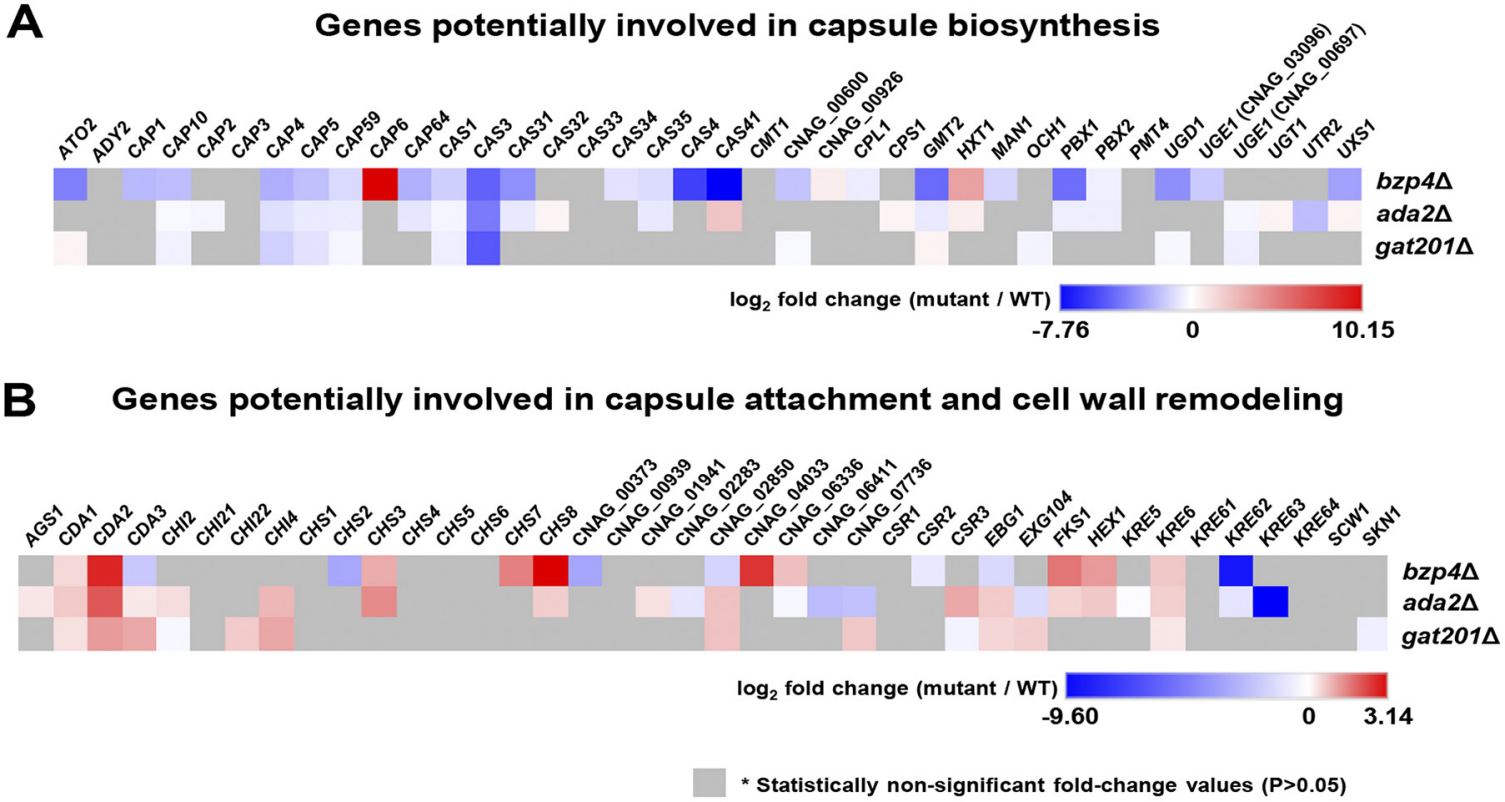
Heatmap of known capsule-regulating and cell wall-remodeling genes in *bzp4*Δ, *ada2*Δ, and *gat201*Δ mutants. Heatmap showing transcriptome gene expression values for genes already known to be involved in capsule biosynthesis (A), capsule attachment, and cell wall remodeling (B). The heatmap was constructed using Morpheus (Broad Institute, Cambridge, MA).

Next, we addressed how Bzp4, Ada2, and Gat201 control the expression of genes involved in capsule biosynthesis, attachment, and cell wall remodeling (Fig. 7). More than 77 proteins involved in these processes have been identified (4). We found that deletion of *BZP4*, *ADA2*, or *GAT201* generally decreased the expression of capsule biosynthesis-related genes but did not affect the expression of capsule attachment and cell wall remodeling-related genes under capsule-inducing conditions (LIT medium) (Fig. 7). Interestingly, despite the more critical roles of Gat201 in capsule production than those of Bzp4 and Ada2, Bzp4 regulated more capsule biosynthesis genes than Ada2 and Gat201 (Fig. 7A). In particular, *CAS3* was downregulated in the *bzp4*Δ, *ada2*Δ, and *gat201*Δ mutants (Fig. 7A). *CAS3* is a capsule-related protein involved in the formation of GXM; therefore, a decrease in gene expression would have decreased capsule thickness in the mutant strains. In contrast, the expression of two chitin deacetylase genes, *CDA1* and *CDA2*, was commonly regulated in the *bzp4*Δ, *ada2*Δ, and *gat201*Δ mutants (Fig. 7B), indicating that these three TFs may play a role in chitin and chitosan homeostasis in C. neoformans. It has been previously reported that decreased chitosan levels are connected to increased capsule production (27). Collectively, these data further suggest that Bzp4, Ada2, and Gat201 govern capsule formation by C. neoformans by positively and negatively regulating the expression of various capsule biosynthesis and chitin/chitosan synthesis genes, respectively.

## DISCUSSION

**FIG 8:**
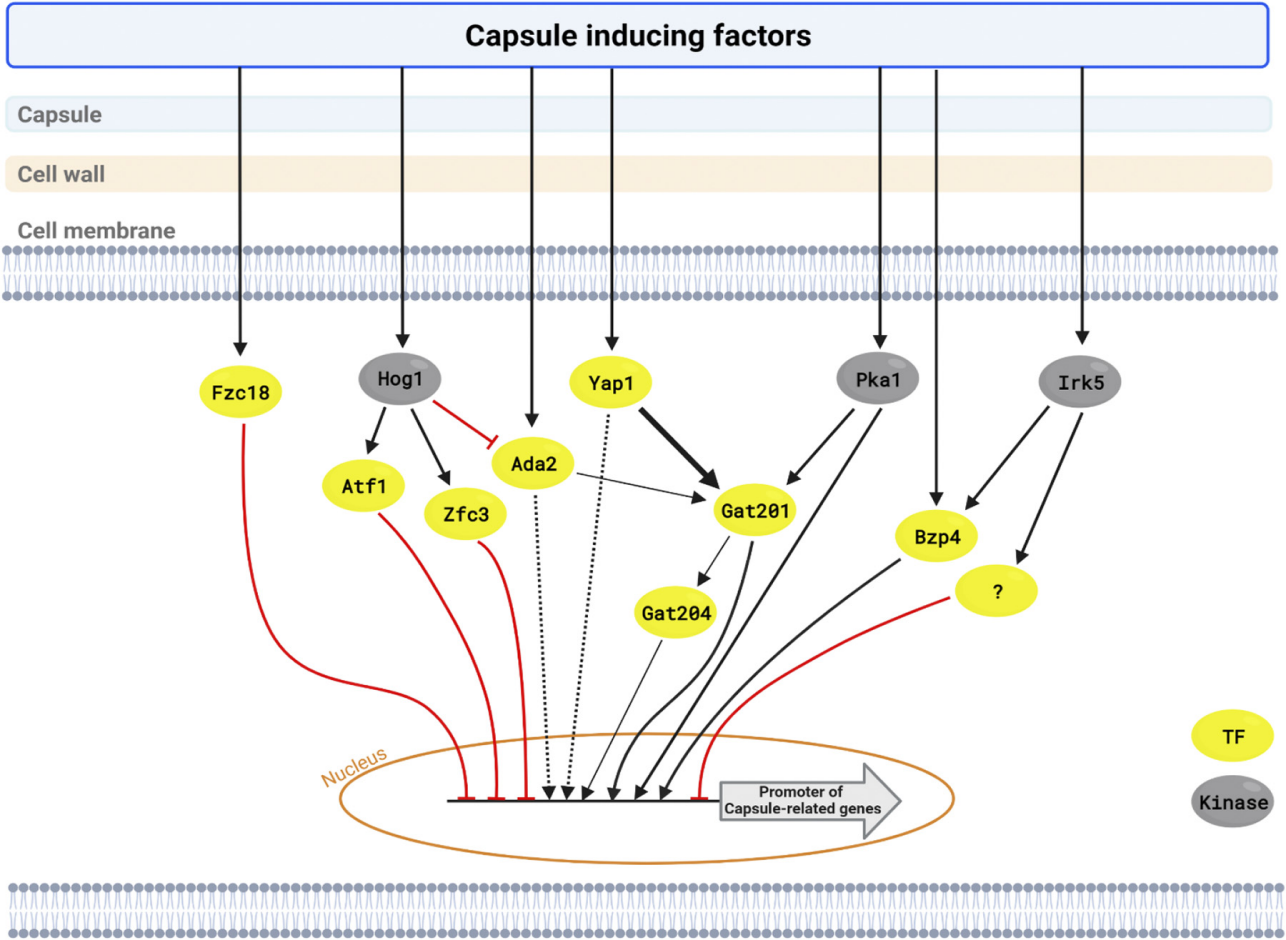
Putative epistatic model of capsule production pathways in C. neoformans. Capsule-inducing conditions control various transcription factors and kinases. Core capsule-regulating transcription factors are regulated by several kinases involved in capsule formation. Yellow circle, TF; silver circle, kinase; black arrow, expression; red arrow, inhibition.

In this study, we elucidated complex signaling networks that regulate the production of the capsule, a key virulence factor for C. neoformans (Fig. 8). We found that four core TFs, Gat201, Yap1, Bzp4, and Ada2, play pivotal roles in the induction of *CAP* (*CAP10*, *CAP59*, *CAP60*, and *CAP64*) and other capsule-related genes. Notably, Yap1 and Ada2 function upstream of Gat201, whereas Bzp4 and Gat201 regulate capsule production independently. As upstream regulators, Pka1 and Irk5 regulate the induction of *GAT201* and *BZP4*, respectively, under capsule induction conditions, whereas Hog1 negatively regulates Ada2.

Based on its significant role in capsule biosynthesis, the role of the four capsule-regulating TFs in the pathogenicity of C. neoformans has been previously reported (12, 14). The *ada2*Δ mutant showed reduced virulence in a murine model of systemic cryptococcosis (28) and *yap1*Δ mutants showed significantly reduced virulence in an insect-killing model (22). Deletion of *GAT201* reduces the virulence and infectivity of C. neoformans (22). However, other virulence factors could contribute to the reduced virulence and/or infectivity of the *ada2*Δ, *yap1*Δ, and *gat201*Δ mutants. Notably, all of these mutants show reduced resistance to oxidative, osmotic, and membrane stress (22, 26). Bzp4 is positively involved in the production of two major virulence factors, melanin and capsule, but is dispensable for general stress resistance, virulence in the insect host, and murine infectivity (25). However, its pathogenic potential has not been fully examined in a classical murine-based virulence assay. One possibility is that the residual capsule and melanin levels in the *bzp4*Δ mutant are sufficient to maintain its virulence. Another possibility is that other unidentified virulence factor(s) are enhanced in the *bzp4*Δ mutant. These possibilities need to be investigated in future studies.

Among the capsule-inducing core TFs, Gat201, a GATA family TF, appears to play the most important role, because its deletion led to the most dramatic decrease in capsule production. Yap1 and Ada2 are likely two major upstream regulators of Gat201, because deletion of both *YAP1* and *ADA2* almost abolished *GAT201* induction, *GAT201* overexpression completely rescued capsule defects in *YAP1* and *ADA2* deletion mutants, and the *yap1*Δ *ada2*Δ mutant was defective in capsule production, like the *gat201*Δ mutant. Supporting this, the transcriptome profiles of the *gat201*Δ and *ada2*Δ mutants under capsule-inducing conditions were highly correlated. Yap1 is a bZIP-containing AP-1-like TF that is generally involved in oxidative stress response (26). Ada2 is a member of the Spt-Ada-Gcn5 acetyltransferase complex which regulates the transcription of stress response genes via histone acetylation, collaborating with other TFs (29). Notably, deletion of Pka1 significantly reduced the induction level of *GAT201*, but not those of *YAP1* and *ADA2*, suggesting that the cAMP/PKA pathway may also independently control the Gat201-dependent pathway. These data also suggest that the marked capsule defects observed in the cAMP/PKA mutants are likely caused by the reduced expression of Gat201. RNAseq-based transcriptome analysis revealed several additional TFs downstream of Gat201, including Gat204 (Data Set S1 and Fig. S7). *gat204*Δ mutants exhibited fewer defects in capsule production than *gat201*Δ mutants (Fig. S7), suggesting that Gat204 likely governs the subset of Gat201-dependent genes, consistent with previously reported data (30).

The basal *ADA2* expression levels increased in the *hog1*Δ mutant, indicating that Hog1 negatively regulates Ada2, consistent with our previous microarray data, which also reported that Hog1 positively regulates Atf1 and Zfc3 (31). Supporting this, deletion of *HOG1* markedly increased capsule production (21). However, during capsule-inducing conditions, *ADA2* induction levels were almost equivalent in both the wild-type and *hog1*Δ mutant strains, and capsule induction of *atf1*Δ and *zfc3*Δ increased under some capsule-inducing conditions, implying that Hog1 controls capsule production through downstream factors such as Atf1, Zfc3, and Ada2.

Our results showed that Bzp4 positively regulated capsule production in a Gat201/Yap1/Ada2-independent manner. The induction of *BZP4* during capsule induction is positively controlled by Irk5 (infectivity-related kinase 5). Because deletion of *IRK5* led to highly increased capsule production, other downstream TFs besides Bzp4 work downstream of Irk5. Our previous analysis of the melanin-regulating signaling network revealed that the homeobox TF Hob1 is the major upstream regulator of Bzp4 in melanin production (25). However, the fact that the *hob1*Δ mutant is not defective in capsule production suggests that Bzp4 is controlled by different upstream regulators depending on external signals. Nevertheless, the fact that all these signaling components, Irk5, Hob1, and Bzp4, are not evolutionarily conserved in other fungi suggests that this signaling pathway has been uniquely developed in C. neoformans. Although Gat201 and Bzp4 are independently controlled, it is possible that these two TFs cooperate to regulate capsule production, because the deletion of *BZP4* did not further decrease capsule production in the *gat201*Δ mutant. Future studies must focus on how Gat201 and Bzp4 cooperate in capsule production.

Our RNA-seq data showed that Bzp4, Gat201, and Ada2 controlled the expression of genes involved in diverse primary and secondary metabolite pathways and capsule synthesis and attachment processes. In general, genes involved in capsule biosynthesis were positively regulated by the three TFs, whereas those involved in capsule attachment and cell wall remodeling, such as chitin/chitosan synthesis genes, were negatively regulated. The finding that Bzp4 positively regulated the expression of a larger number of capsule biosynthesis genes than Gat201 was unexpected, because capsule defects were more pronounced in the *gat201*Δ mutant than in the *bzp4*Δ mutant. Therefore, in addition to controlling the expression of capsule biosynthesis genes, Gat201 may also indirectly affect capsule production. The role of Bzp4, Gat201, and Ada2 in the expression of chitin and chitosan synthesis genes also appeared to be associated with capsule defects in TF mutants. In C. neoformans, Chs3 (chitin synthase 3) produces chitin that is subsequently converted to chitosan (4). Reportedly, the deletion of two chitin deacetylase genes, *CDA1* and *CDA2*, or *CHS3* increased chitin levels but decreased chitosan levels and enhanced capsule production (27). This may be because increased chitosan masks the capsule attachment sites and inhibits encapsulation (4). Our findings that *CDA1* and *CDA2* expression were upregulated in *bzp4*Δ, *gat201*Δ, and *ada2*Δ mutants and *CHS3* expression was upregulated in *bzp4*Δ and *ada2*Δ mutants support this idea. However, the quantification of chitosan levels in the wild-type and capsule-defective TF mutant strains showed no significant differences (Fig. S8). Therefore, differential chitosan levels are unlikely to contribute to the reduced capsule production in TF mutants. Nevertheless, we cannot exclude the possibility of the involvement of Bzp4, Gat201, and Ada2 in capsule attachment and cell wall remodeling. This topic should be further studied in the future.

In conclusion, we demonstrated that Bzp4, Ada2, Yap1, and Gat201 are core TFs which play pivotal roles in capsule production in C. neoformans. Because the capsule is one of the major virulence factors in C. neoformans, therapeutic strategies targeting these signaling pathways could be a reasonable option for the development of anti-cryptococcal drugs. Bzp4 and Gat201 could be better therapeutic targets because they are more evolutionarily divergent than other TFs and are the two major capsule-regulating TFs. For this, the upstream and downstream networks of Bzp4 and Gat201 need to be functionally characterized in future studies.

## MATERIALS AND METHODS

### Strains of *Cryptococcus neoformans* and capsule induction conditions.

The C. neoformans strains used in this study are listed in Table S1. The strains were cultured and maintained in yeast extract-peptone-dextrose medium. For the capsule production assay, the strains were inoculated into 2 mL of YPD broth and cultured overnight at 30°C in a shaking incubator. Cells were spun down, washed twice with PBS, and resuspended in 1 mL PBS. Each strain was spotted (5 μL) on Dulbecco’s modified Eagle’s medium, Littman’s medium, and 10% fetal bovine serum agar medium. Cells were further incubated for 48 h at 37°C. After incubation, the capsule was visualized by staining with India ink and observed under a microscope. Quantitative measurement of capsule size was performed by microscopically measuring the diameters of the capsule and cell using SPOT Advanced version 4.6 software.

### Gene disruption and complementation.

The gene deletion mutants used in this study were produced as follows. L1-L2 and R1-R2 primer pairs (Table S1) were used in the first round of PCR. The *NEO* selection marker was amplified with M13 forward extended (M13Fe) and M13 reverse extended (M13Re) primers using a plasmid containing the *NEO* gene as a template. The first-round PCR products of the flanking regions and *NEO* markers were purified together and used as the templates for the second double joint-PCR (DJ-PCR). In the second round of PCR, the 5′- and 3′-gene disruption cassettes containing split *NEO* selection markers were amplified using L1-split primer 2 and R2-split primer 1, respectively (Table S1). To perform biolistic transformation, background strains were cultured in YPD medium at 30°C in a shaking incubator for 16 h. The cultured cells were centrifuged, spread on YPD agar medium containing 1 M sorbitol, and further incubated at 30°C for 4 h. The dried disruption cassettes were deposited onto 0.6-μm gold micro carrier beads (Bio-Rad), delivered into the cells on YPD + 1 M sorbitol agar plates using a particle delivery system (PDS-100, Bio-Rad), and incubated at 30°C for 4 h. Subsequently, the incubated cells were recovered using a scraper, spread on YPD agar medium containing 50 μg/mL G418, and incubated at 30°C until colonies appeared on plates. G418-resistant colonies were picked and restreaked on fresh YPD + G418 plates. Positive transformants were confirmed using diagnostic PCR with B79-SO primers and verified using Southern blot analysis.

### Construction of complemented strains.

To verify the phenotypes of the *bzp4*Δ, *gat201*Δ, *yap1*Δ mutants, the *bzp4*Δ::*BZP4-mChrry*, *gat201*Δ::*GAT201-GFP*, and *yap1*Δ::*YAP1-GFP* complemented strains were constructed as follows. First, the full-length *BZP4*, *GAT201*, and *YAP1* genes were amplified using PCR with H99 genomic DNA as the template and directly cloned into the TOPO vector (Invitrogen) to generate the plasmids pTOP-BZP4, pTOP-GAT201, and pTOP-YAP1. After confirmation of the DNA sequence, the *BZP4*, *GAT201*, and *YAP1* inserts were subcloned into the plasmid pNEO-RFPhog1t (YSBE524) or pNEO-GFPhog1t (YSB508) to produce the plasmids pNEO-BZP4-mCherry, pNEO-GAT201-GFP, and pNEO-YAP1-GFP. For targeted reintegration of the *BZP4*-*mCherry*, *GAT201-GFP*, and *YAP1-GFP* alleles into their native loci, pNEO-BZP4-mCherry, pNEO-GAT201-GFP, and pNEO-YAP1-GFP were linearized by AflII, MfeI, and PmlI (New England Biolabs) digestion and introduced into the *bzp4*Δ, *gat201*Δ, and *yap1*Δ mutants using biolistic transformation. The correct genotype of the *bzp4*Δ::*BZP4-mChrry*, *gat201*Δ::*GAT201-GFP*, and *yap1*Δ::*YAP1-GFP* complemented strains was confirmed using diagnostic PCR.

### Construction of GAT201 overexpression strains.

To generate the *GAT201* constitutive overexpression strain, the native promoter of *GAT201* was replaced with the histone *H3* promoter using an amplified homologous recombination cassette (Fig. S2C). The primer pairs B17609, B17610 and B17611, B17612 were used for amplification of the native promoter and 5′ coding regions (from ATG), respectively, of *GAT201* in the first round of PCR. The hygromycin resistance gene (*HYG*)-*H3* promoter region was amplified with the primer pair B4017, B4018. In the second-round PCR, the 5′ region of the P*_H3_*:*GAT201* cassette was amplified by DJ-PCR using the mixed templates of the native promoter region of *GAT201* and the 5′ region of the *HYG*-*H3* promoter region with the primer pair B17609, B5752 (Table S1). The 3′ region of the P*_H3_*:*GAT201* cassette was similarly amplified by using the mixed templates of the 5′ coding region of *GAT201* and the 3′ region of the *HYG*-*H3* promoter region with the primer pair B5751, B17612. Next, the combined split P*_H3_*:*GAT201* cassettes were introduced into the wild-type (H99S), *yap1*Δ (YSB815), *ada2*Δ (YSB2381), and *yap1*Δ *ada2*Δ (YSB6054) strains by biolistic transformation. Stable transformants were selected on YPD medium containing 300 μg/mL hygromycin B. Positive transformants were confirmed using diagnostic PCR with a primer pair (B2931/B79).

### Capsule production assay.

For capsule induction, each strain was incubated for 16 h at 30°C in liquid YPD medium. The cultured cells were washed with PBS, spotted (5 μL) onto DMEM, LIT, or 10% FBS solid medium and further incubated for 48 h at 37°C. After incubation, the capsule was visualized by staining with India ink and observed under a microscope. Quantitative measurement of capsule size was performed by microscopically measuring the diameters of the capsule and cell using SPOT Advanced version 4.6 software.

### Assessment of cellular localization of tagged strains under capsule-inducing conditions.

*bzp4*Δ::*BZP4-mCherry* (YSB5408), *gat201*Δ::*GAT201*-GFP (YSB6745), *yap1*Δ::*YAP1*-GFP (YSB2723), and *ADA2*-GFP (YSB3405) were cultured in 50 mL of YPD liquid medium at 30°C in a shaking incubator for 16 h and subcultured in fresh YPD liquid medium until the optical density at 600 nm (OD_600_) reached 0.6 to 0.8. Subcultured cells were washed with PBS and suspended in 40 mL LIT medium. After suspension, cells were incubated in a 37°C shaking incubator, a portion (1 mL) was sampled at each time point (1, 2, and 4 h), and the cells were fixed in paraformaldehyde. Fixed cells were stained with Hoechst for nuclear staining and observed using differential interference contrast (DIC) and fluorescence microscopy.

### Total RNA preparation and qRT-PCR.

The WT and mutant strains were inoculated into 50 mL of YPD broth and cultured overnight at 30°C in a shaking incubator. Cells were subcultured in 80 mL of fresh YPD broth until the OD_600_ reached 0.6 to 0.8. The subcultured cells were then washed with PBS and suspended in 40 mL of LIT liquid medium. After suspension, the cells were incubated in a 37°C shaking incubator for 2 h, centrifuged, frozen in liquid nitrogen, and lyophilized. Total RNA was isolated via the TRIzol extraction method using Easy-Blue (Intron). Total RNA from each strain was purified using an RNeasy kit (Qiagen). cDNA was synthesized with reverse transcriptase (Thermo Fisher Scientific) using purified total RNA. Gene (*CAP10*, *CAP59*, *CAP60*, *CAP64*, *GAT201*, *YAP1*, *BZP4*, and *ADA2*) expression was analyzed using qRT-PCR with specific primer pairs (Table S1) and the CFX96TM Real-Time system (Bio-Rad). *ACT1* expression was used as a normalization control. Statistical differences between samples were analyzed using a Student’s *t* test.

### RNA-seq and data analysis.

Transcriptome patterns of the H99S, *bzp4*Δ, *ada2*Δ, and *gat201*Δ strains were comparatively analyzed using RNA sequencing. C. neoformans strains were grown at 30°C for 16 h in YPD medium, transferred to 50 mL of fresh YPD medium, and incubated at 30°C until the OD_600_ reached 0.8. Half of the cells were further incubated in LIT liquid medium for 2 h. Cells were harvested using centrifugation and lyophilized. Total RNAs was prepared as described above and purified using an RNeasy minikit (Qiagen). The concentration was measured using Quant-IT RiboGreen (Invitrogen). RNA quality was verified using a TapeStation RNA Screentape (Agilent). Cultured samples were prepared independently thrice. A cDNA library was constructed with 1 μg total RNAs for each sample using the Illumina TruSeq mRNA library kit (Illumina) and sequenced on the Illumina platform. The adapter sequences were trimmed from the sequencing reads using Cutadapt v3.4 with Python 3.7.4. (32). The reference genome sequence of C. neoformans H99 and annotation data derived from the Broad Institute (Cambridge, MA) were downloaded from the NCBI FTP server. The reads were aligned to the C. neoformans H99 genome sequence using Hisat2 v2.2.1, with the HISAT and Bowtie2 algorithm, and processed as previously described (33). Hisat2 was performed using the “-p 30” and “– dta -1” options, with the other parameters set to default. Aligned reads were converted and then sorted using Samtools v1.9 (34) with the “-Sb -@ 8” option for converting, the “-@ 20 -m 2000000000” option for sorting, and the other parameters set to default. Transcript assembly and abundance estimation were performed using Stringtie v2.1.1 with the “-p 12” and “-B” options to run the Ballgown analysis (35). The assembled transcripts were merged into a single GTF file, and the relative transcript abundance was calculated using fragments per kilobase of exon per million fragments mapped (FPKM). The FPKM and read-count matrix were generated using the R package “isoformswitchanalyzerR” and analyzed using DESeq2 (36, 37). Differentially expressed gene (DEG) analysis was performed using DESeq2 v1.24.0 with default settings using Ballgown. The volcano plot was illustrated using R v4.1.0 and the R package “EnhancedVolcano” (38) with a cutoff of more than 2-fold change (*P* < 0.05). A PCA plot was generated using the R package “DeBrowser” and a heatmap was generated using the R package (39). KEGG pathway analysis was performed using the R package “Cluster profiler” (40) after changing the H99S gene locus ID to JEC21 ortholog locus ID. The list of differentially expressed genes is provided in Data Set S1 in the supplemental material.

### Measurement of chitosan content in cell walls.

The chitosan content of the wild-type and mutant cells was quantified using a 3-methyl-2-benzothiazolinone hydrazone (MBTH)-based chemical assay with some modifications (41, 42). Cells were cultured in 50 mL liquid YPD in a shaking incubator for 48 h at 30°C, collected by centrifugation, washed three times with PBS (pH 7.5), frozen in liquid nitrogen, and lyophilized. Lyophilized cells were resuspended in 6% KOH, incubated at 80°C for 30 min with occasional tapping to eliminate nonspecific MBTH-reactive molecules, washed three times with PBS (pH 7.5) to neutralize the pH, resuspended in PBS, sonicated for 30 s, and adjusted to a final concentration of OD_600_ = 1.0. For the MBTH assay, 0.1 mL of the sample was mixed with an equal volume of 1 M HCl, heated at 110°C for 2 h, transferred to a 25°C water bath for cooling, and supplemented with 2.5% NaNO_2_. Samples were incubated at room temperature for 15 min, supplemented with 200 mL of 12.5% ammonium sulfamate, and incubated for another 5 min at room temperature. Next, 200 mL of 0.25% MBTH was added to each sample, followed by 30 min incubation at 37°C, addition of 200 mL 0.5% FeCl_3_, and another 5 min incubation at 37°C. Finally, the optical density of each sample was measured at 595 nm.

### Data availability.

RNA-seq data have been deposited in the Gene Expression Omnibus (GEO) database (accession no. GSE208163). We will provide any strains and materials used in this study upon request.
