# Supplementary material for: Unraveling Capsule Biosynthesis and Signaling Networks in Cryptococcus neoformans
Source: Microbiol Spectr. 2022 Oct 26;10(6):e02866-22. doi: 10.1128/spectrum.02866-22 (PMC9769619; doi:10.1128/spectrum.02866-22)
Supplement: Supplemental file 1 — Table S1 and Fig. S1 to S8. Download spectrum.02866-22-s0001.pdf, PDF file, 2.9 MB [file spectrum.02866-22-s0001.pdf]

SUPPLEMENTAL MATERIAL

Table S1. *C. neoformans* strains and primers used in this study

C. neoformans strains used in this study			
**Strain**	**Genotypes**	**Parents**	**References**
H99S	*MAT*α		(1)
YSB64	*MAT*α *hog1*Δ::*NAT-STM#177*	H99S	(2)
YSB188	*MAT*α *pka1*Δ::*NAT-STM#191*	H99S	(2)
YSB349	*MAT*α *skn7*::*NAT-STM#201*	H99S	(3)
YSB488	*MAT*α *mbs1*Δ::*NAT-STM#150*	H99S	(3)
YSB501	*MAT*α *fzc34*Δ::*NAT-STM#231*	H99S	(3)
YSB510	*MAT*α *fzc1*Δ::*NAT-STM#116*	H99S	(3)
YSB552	*MAT*α *ire1*Δ::*NAT-STM#224*	H99S	(2)
YSB676	*MAT*α *atf1*::*NAT-STM#220*	H99S	(3)
YSB718	*MAT*α *fzc29*::*NAT-STM#225*	H99S	(3)
YSB774	*MAT*α *fzc24*::*NAT-STM#292*	H99S	(3)
YSB815	*MAT*α *yap1*::*NAT-STM#296*	H99S	This study
YSB1074	*MAT*α *fzc33*Δ::*NAT-STM#43*	H99S	(3)
YSB1099	*MAT*α *bzp3*Δ::*NAT-STM#146*	H99S	(3)
YSB1106	*MAT*α *crl6*Δ::*NAT-STM#231*	H99S	(3)
YSB1172	*MAT*α *mln1*Δ::*NAT-STM#146*	H99S	(3)
YSB1209	*MAT*α *fzc46*Δ::*NAT-STM #177*	H99S	(3)
YSB1290	*MAT*α *yap1*::*NAT-STM#296*	H99S	This study
YSB1302	*MAT*α *mcm1*Δ::*NAT-STM#218*	H99S	(3)
YSB1311	*MAT*α *gat204*Δ::*NAT-STM#218*	H99S	(3)
YSB1339	*MAT*α *fkh2*Δ::*NAT-STM#219*	H99S	(3)
YSB1396	*MAT*α *clr1*Δ::*NAT-STM#242*	H99S	(3)
YSB1435	*MAT*α *hob4*Δ::*NAT-STM#159*	H99S	(3)
YSB1464	*MAT*α *usv101*Δ::*NAT-STM#191*	H99S	(3)
YSB1585	*MAT*α *hob5*Δ::*NAT-STM#219*	H99S	(3)
YSB1592	*MAT*α *jjj1*Δ::*NAT-STM#240*	H99S	(3)
YSB1834	*MAT*α *clr3*Δ::*NAT-STM#102*	H99S	(3)
YSB1842	*MAT*α *fzc51*Δ::*NAT-STM#159*	H99S	(3)
YSB1846	*MAT*α *fzc14*Δ::*NAT-STM#43*	H99S	(3)
YSB1850	*MAT*α *hcm1*Δ::*NAT-STM#177*	H99S	(3)
YSB1894	*MAT*α *bzp4*Δ::*NAT-STM#295*	H99S	This study
YSB1895	*MAT*α *bzp4*Δ::*NAT-STM#295*	H99S	This study
YSB1898	*MAT*α *rds2*Δ::*NAT-STM#242*	H99S	(3)
YSB1969	*MAT*α *bud32*Δ::*NAT-STM#296*	H99S	(2)
YSB2001	*MAT*α *hob3*Δ::*NAT-STM#211*	H99S	(3)
YSB2108	*MAT*α *zfc3*Δ::*NAT-STM#232*	H99S	(3)

YSB2134	*MATα zap104Δ::NAT-STM#204*	H99S	(3)
YSB2171	*MATα fzc49Δ::NAT-STM#5*	H99S	(3)
YSB2211	*MATα liv1Δ::NAT-STM#213*	H99S	(3)
YSB2221	*MATα fzc45Δ::NAT-STM#58*	H99S	(3)
YSB2231	*MATα zfc4Δ::NAT-STM#210*	H99S	(3)
YSB2244	*MATα hlh4Δ::NAT-STM#295*	H99S	(3)
YSB2250	*MATα fzc17Δ::NAT-STM#240*	H99S	(3)
YSB2295	*MATα hsf2Δ::NAT-STM#205*	H99S	(3)
YSB2320	*MATα fzc18Δ::NAT-STM#212*	H99S	(3)
YSB2326	*MATα fzc16Δ::NAT-STM#212*	H99S	(3)
YSB2329	*MATα hlh3Δ::NAT-STM#208*	H99S	(3)
YSB2381	*MATα ada2Δ::NAT-STM#232*	H99S	This study
YSB2382	*MATα ada2Δ::NAT-STM#232*	H99S	This study
YSB2447	*MATα fzc30Δ::NAT-STM#230*	H99S	(3)
YSB2481	*MATα hap1Δ::NAT-STM#240*	H99S	(3)
YSB2493	*MATα sre1Δ::NAT-STM#240*	H99S	(3)
YSB2723	*MATα yap1Δ::YAP1-GFP-NEO*	YSB1290	This study
YSB2952	*MATα irk5Δ:: NAT-STM#213*	H99S	(2)
YSB3026	*MATα hob7Δ::NAT-STM#159*	H99S	(3)
YSB3096	*MATα nrg1::NAT-STM#123*	H99S	(3)
YSB3300	*MATα gat201::NAT-STM#273*	H99S	This study
YSB3301	*MATα gat201::NAT-STM#273*	H99S	This study
YSB3405	*MATα ADA2-GFP*	H99S	This study
YSB3699	*MATα cdc2801Δ::NAT-STM#191*	H99S	(2)
YSB3714	*MATα pos5Δ::NAT-STM#58*	H99S	(2)
YSB4081	*MATα cap10Δ::NEO*	H99S	This study
YSB5408	*MATα bzp4Δ::BZP4* p*NEO-mCherry*	YSB1895	This study
YSB5499	*MATα bzp4Δ::BZP4* p*NEO*	YSB1895	This study
YSB6052	*MATα gat201Δ::NAT bzp4Δ::NEO*	YSB3300	This study
YSB6054	*MATα yap1Δ::NAT ada2Δ::NEO*	YSB815	This study
YSB6055	*MATα yap1Δ::NAT bzp4Δ::NEO*	YSB815	This study
YSB6167	*MATα gat201Δ::NAT ada2Δ::NEO*	YSB3300	This study
YSB6169	*MATα ada2Δ::NAT bzp4Δ::NEO*	YSB2382	This study
YSB6695	*MATα yap1Δ::NAT gat201Δ::NEO*	YSB815	This study
YSB6745	*MATα gat201Δ::GAT201-GFP-NEO*	YSB3300	This study
YSB10417	*MATα* P_{H3}:*GAT201::HYG*	H99S	This study
YSB10418	*MATα* P_{H3}:*GAT201::HYG yap1Δ::NAT*	YSB815	This study
YSB10419	*MATα* P_{H3}:*GAT201::HYG ada2Δ::NAT*	YSB2381	This study
YSB10420	*MATα* P_{H3}:*GAT201::HYG yap1Δ::NAT ada2Δ::NEO*	YSB6054	This study

**Primers used in this study**		
**Name**	**Primer description**	**Sequence (5' to 3')**
B2927	*GAT201* 5' flanking region primer 1	TCCGTTGAGATAGCGTTG
B2928	*GAT201* 5' flanking region primer 2	TCACTGGCCGTCGTTTTACATGGTGGAGGTGTAGGACTG
B2929	*GAT201* 3' flanking region primer 1	CATGGTCATAGCTGTTTCCTGCTCAACCACCTTTTTGTGC
B2930	*GAT201* 3' flanking region primer 2	GGGAATCTGGTGTCAGTATTG
B2931	*GAT201* diagnostic screening primer, pairing with B79	AACCTTGCCTAACAACCC
B2932	*GAT201* Southern blot probe primer 1	GTCGGAATGACAAAGGAGATAC
B2933	*GAT201* Southern blot probe primer 2	GGGGGAATAAAGGATAATGC
B2183	*ADA2* 5' flanking region primer 1	GGATGATGGAATCGTATGC
B2184	*ADA2* 5' flanking region primer 2	TCACTGGCCGTCGTTTTACATTATCCACCCTCGGCTTC
B2185	*ADA2* 3' flanking region primer 1	CATGGTCATAGCTGTTTCCTGGCGAACGGTATGACAACTATG
B2186	*ADA2* 3' flanking region primer 2	GGACGAAAACATTGCTCTCAAC
B2182	*ADA2* diagnostic screening primer, pairing with B79	TCAGCAGCAGCAGGTAAAC
B9851	*ADA2* Southern blot probe primer 1	GAGGATGATGGAATCGTATG
B9852	*ADA2* Southern blot probe primer 2	CAAAAGCAAGTTGGACAGAG
B3738	*BZP4* 5' flanking region primer 1	CGCCCTTTCTATTGTTACAC
B3739	*BZP4* 5' flanking region primer 2	TCACTGGCCGTCGTTTTACGATGACAGGAGGGATGAATC
B3740	*BZP4* 3' flanking region primer 1	CATGGTCATAGCTGTTTCCTGGGGAGAATAACGACTCAATGT C
B3741	*BZP4* 3' flanking region primer 2	TCATTGCTGACTGGGAAG
B3736	*BZP4* diagnostic screening primer, pairing with B79	AAAGAGGCGGTGTTGAAG
B3737	*BZP4* Southern blot probe primer	AGCCAGGTAATCTTGGAGG
B1586	*YAP1* 5' flanking region primer 1	TTGCTGCGATGTGGTCTTG
B1587	*YAP1* 5' flanking region primer 2	GCTCACTGGCCGTCGTTTTACACAAAGGGTCAACAAGGG
B1588	*YAP1* 3' flanking region primer 1	ATCTTGATGGAGGGGGGTTGG
B1589	*YAP1* 3' flanking region primer 2	CATGGTCATAGCTGTTTCCTGAACGACGACCATCGCAGTAG
B1590	*YAP1* diagnostic screening primer, pairing with B79	TGGTGCTCAAGAGGGAAGTTAG
B2871	*YAP1* Southern blot probe primer	CTACTCTGTGGTGGCGTAAG
B9966	*GAT201* LP (*GFP* tagging)	CTCGAGCGTTTCCTATTCCTGTTGTG
B9967	*GAT201* RP (*GFP* tagging)	GCGGCCGCAGGAAGGAAAGCACTTGGTAAC
B9968	*GAT201* sequencing primer 1 (*GFP* tagging)	CTATGCATTGGATCAATTG
B9969	*GAT201* sequencing primer 2 (*GFP* tagging)	CCATACTCCCGCCCGCAAC
B9970	*GAT201* sequencing primer 3 (*GFP* tagging)	CGGGAATTGGTGTCGCATC
B10107	*GAT201* screening primer (*GFP* tagging)	TCATCCGCCTAAATGTCC
B3148	*YAP1* LP (*GFP* tagging)	GGATCCATGCAGTCCGCACTCACCCC
B3149	*YAP1* RP (*GFP* tagging)	GGATCCGGTGCTCAAGAGGGAAGTTAG

B3152	*YAP1* sequencing primer 1 (*GFP* tagging)	CCAACACGGCCGCGTTCCTCG
B3153	*YAP1* sequencing primer 2 (*GFP* tagging)	TGAAGGTGATGGAAAAAGAGA
B3154	*YAP1* sequencing primer 3 (*GFP* tagging)	TCATCCCCTTCCATTTCC
B3155	*YAP1* sequencing primer 4 (*GFP* tagging)	ACTCTGGATGAGATTCGGG
B9346	*YAP1* screening primer (*GFP* tagging)	AGTCGTGGGGATGTTCTTGG
B6514	*ADA2* diagnostic screening primer, pairing with B79 (*GFP* chromosol integration)	CGTCCTCCAGATGAAGAAG
B6515	*ADA2* 5' flanking region primer 1 (*GFP* chromosomal integration)	CAAGAGAGAGGATGCCAAG
B6516	*ADA2* 5' flanking region primer 2 (*GFP* chromosomal integration)	GCTCACAGAGCCACCGCCACCTCCATTGAGCCTAATCTCA
B6517	*ADA2* 3' flanking region primer 1 (*GFP* chromosomal integration)	GCCACTCGAATCCTGCATGCGCAAATTTATAGTCACTATT
B6518	*ADA2* 3' flanking region primer 2 (*GFP* chromosomal integration)	TTTTGGAAGCACCTTGCC
B6628	*ADA2* Southern blot probe primer (*GFP* chromosomal integration)	AAAGCGGATAGCGGAACTC
B9003	*BZP4* LP with XhoI cut site (*mCherry* tagging)	CTCGAGCGCTTTCGCAATGTCAGG
B9004	*BZP4* RP with NotI cut site (*mCherry* tagging)	GCGGCCGCTCTTGACATTGAGTCGTT
B9005	*BZP4* sequencing primer (*mCherry* tagging)	GTTACTGTTACAGCGAAC
B9348	*BZP4* screening primer (*mCherry* tagging)	GCGAGAGTGGTTGGTTAGTG
B1026	M13 Forward extended	GTAAAACGACGGCCAGTGAGC
B1027	M13 Reverse extended	CAGGAAACAGCTATGACCATG
B79	Screening primer	TGTGGATGCTGGCGGAGGATA
B1454	*NAT* split marker primer 1	AAGGTGTTCCCCGACGACGAATCG
B1455	*NAT* split marker primer 2	AACTCCGTCGCGAGCCCCATCAAC
B1886	*NEO* split marker primer 1	TGGAAGAGATGGATGTGC
B1887	*NEO* split marker primer 2	ATTGTCTGTTGTGCCCAG
B5751	*HYG* split marker primer 1	CGAAGAATCTCGTGCTTTC
B5752	*HYG* split marker primer 2	ATTGACCGATTCCTTGCG
B4017	*HYG* Forward extended	GCATGCAGGATTCGAGTG
B4018	*HYG* Reverse extended	GTGATAGATGTGTTGTGGTG
B17609	*GAT201* 5' flanking region primer 1 (*H3* promoter replacement)	GGTCGGAATGACAAAGGAGA
B17610	*GAT201* 5' flanking region primer 2 (*H3* promoter replacement)	GCCACTCGAATCCTGCATGCTGCGTGGGTGGCGCTGTGCT
B17611	*GAT201* 3' flanking region primer 1 (*H3* promoter replacement)	CACCACAACACATCTATCACATGTCAAAGTACTCCCACGA
B17612	*GAT201* 3' flanking region primer 2 (*H3* promoter replacement)	AAAACAGGCAGGTACGGTTG
B679	qRT-PCR primer for *ACT1*	CGCCCTTGCTCCTTCTTCTATG
B680	qRT-PCR primer for *ACT1*	GACTCGTCGTATTCGCTCTTCG
B8640	qRT-PCR primer for *CAP10*	ATTCATTCCCGATTGGCG
B7163	qRT-PCR primer for *CAP10*	GAGAACCAAACAGACGACG

B8684	qRT-PCR primer for *CAP59*	GCTATTAGAGGCTACAAGCG
B8685	qRT-PCR primer for *CAP59*	GGGTGAACAACCTATCGTG
B8643	qRT-PCR primer for *CAP60*	ACGCTATGAACGAAGAGGC
B8644	qRT-PCR primer for *CAP60*	GGAGTGAAAACAGAGTTGGG
B8645	qRT-PCR primer for *CAP64*	CAAGGAAAGGGCATTCAGAG
B8646	qRT-PCR primer for *CAP64*	TCAGAAAGCATTGCCTGG
B9422	qRT-PCR primer for *GAT201*	GGAGTATGGCTGAAATCTG
B6290	qRT-PCR primer for *GAT201*	GGAGTATGGCTGAAATCTGG
B6651	qRT-PCR primer for *YAP1*	CCATGCCCGTTAACAGTCGC
B2871	qRT-PCR primer for *YAP1*	CTACTCTGTGGTGGCGTAAG
B9420	qRT-PCR primer for *BZP4*	TCTTTCCCAAGTAGCATTCCTCG
B9421	qRT-PCR primer for *BZP4*	GCTCGTCATCCCAACTATCAAAAC
B6368	qRT-PCR primer for *ADA2*	TGATGCCGAAATGGCTGTAA
B2187	qRT-PCR primer for *ADA2*	TTCATCTGGAGGACGAGTG

References

1. Perfect JR, Ketabchi N, Cox GM, Ingram CW, Beiser CL. 1993. Karyotyping of *Cryptococcus neoformans* as an epidemiological tool. J Clin Microbiol 31:3305-9.

2. Lee KT, So YS, Yang DH, Jung KW, Choi J, Lee DG, Kwon H, Jang J, Wang LL, Cha S, Meyers GL, Jeong E, Jin JH, Lee Y, Hong J, Bang S, Ji JH, Park G, Byun HJ, Park SW, Park YM, Adedoyin G, Kim T, Averette AF, Choi JS, Heitman J, Cheong E, Lee YH, Bahn YS. 2016. Systematic functional analysis of kinases in the fungal pathogen *Cryptococcus neoformans*. Nat Commun 7:12766.

3. Jung KW, Yang DH, Maeng S, Lee KT, So YS, Hong J, Choi J, Byun HJ, Kim H, Bang S, Song MH, Lee JW, Kim MS, Kim SY, Ji JH, Park G, Kwon H, Cha S, Meyers GL, Wang LL, Jang J, Janbon G, Adedoyin G, Kim T, Averette AK, Heitman J, Cheong E, Lee YH, Lee YW, Bahn YS. 2015. Systematic functional profiling of transcription factor networks in *Cryptococcus neoformans*. Nat Commun 6:6757.

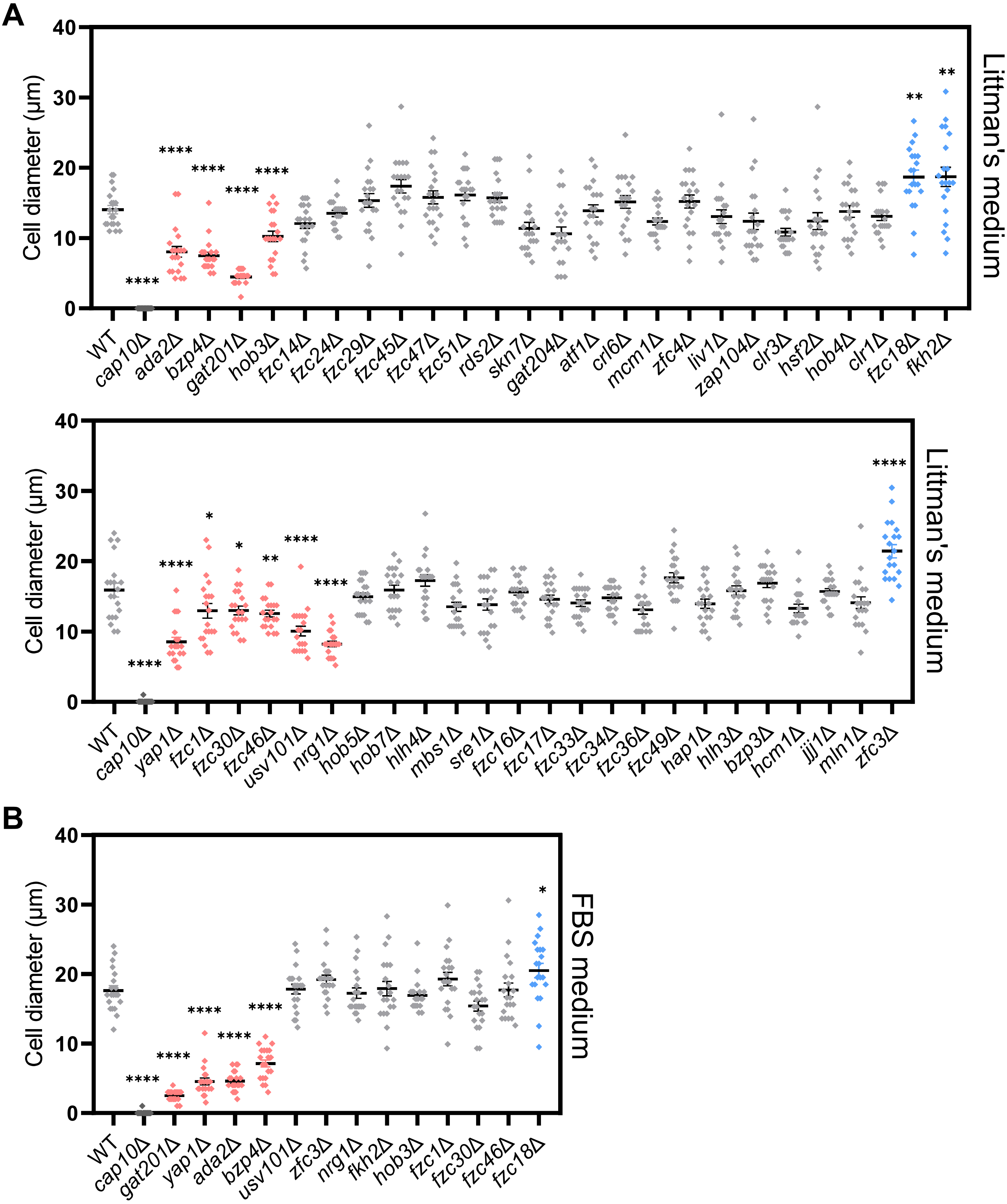

A
Cell diameter (µm)
Littman's medium
WT
cap10Δ
ada2Δ
bzp4Δ
gat201Δ
hob3Δ
fzc14Δ
fzc24Δ
fzc29Δ
fzc45Δ
fzc47Δ
fzc51Δ
rds2Δ
skn7Δ
gat204Δ
atf1Δ
crl6Δ
mcm1Δ
zfc4Δ
liv1Δ
zap104Δ
clr3Δ
hsf2Δ
hob4Δ
clr1Δ
fzc18Δ
fkh2Δ
Cell diameter (µm)
Littman's medium
WT
cap10Δ
yap1Δ
fzc1Δ
fzc30Δ
fzc46Δ
usv101Δ
nrg1Δ
hob5Δ
hob7Δ
hlh4Δ
mbs1Δ
sre1Δ
fzc16Δ
fzc17Δ
fzc33Δ
fzc34Δ
fzc36Δ
fzc49Δ
hap1Δ
hlh3Δ
bzp3Δ
hcm1Δ
jjj1Δ
mln1Δ
zfc3Δ
B
Cell diameter (µm)
FBS medium
WT
cap10Δ
gat201Δ
yap1Δ
ada2Δ
bzp4Δ
usv101Δ
zfc3Δ
nrg1Δ
fkh2Δ
hob3Δ
fzc1Δ
fzc30Δ
fzc46Δ
fzc18Δ

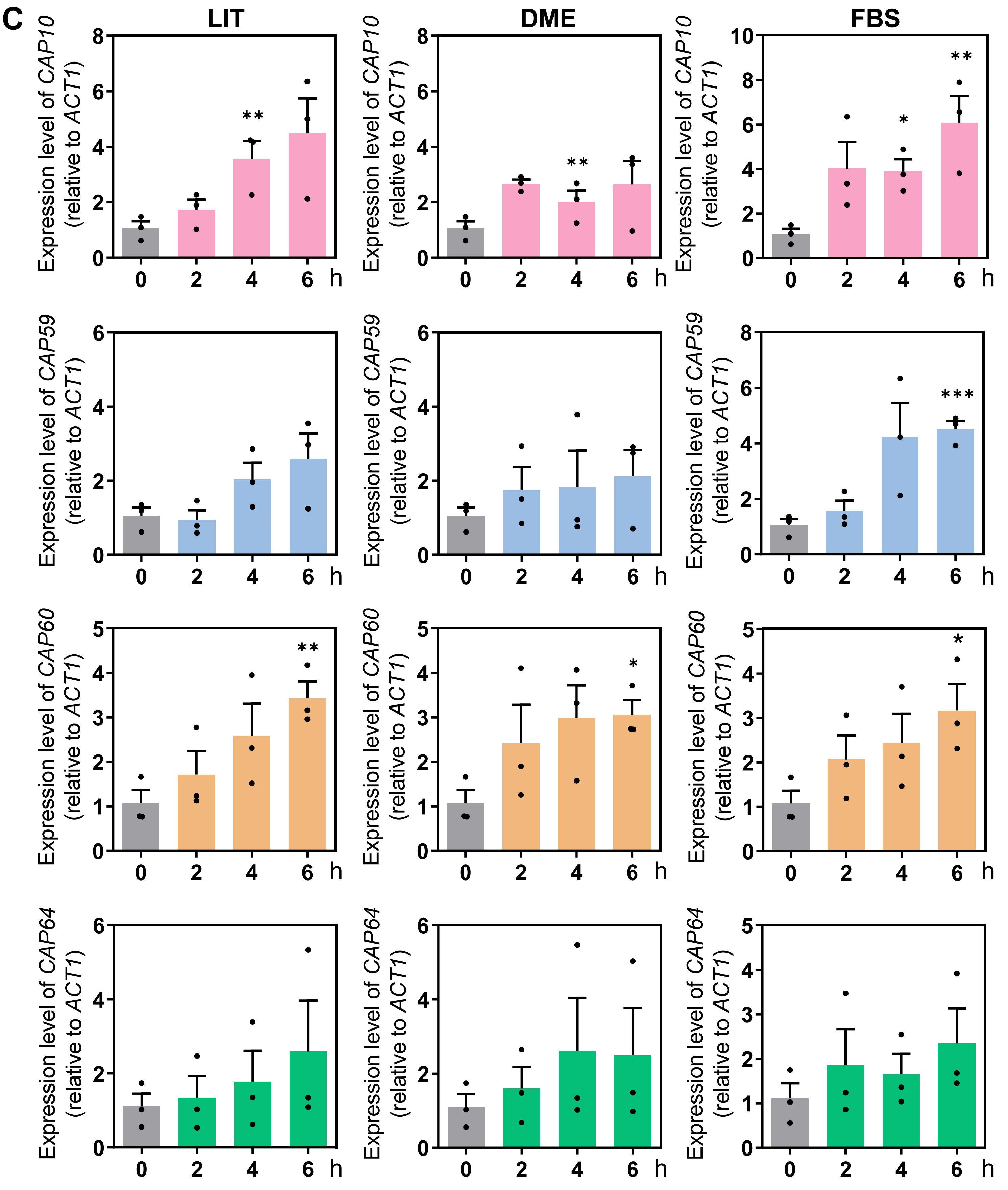

Figure S1. Capsule production of 49 transcription factor mutants in *C. neoformans*. (A) WT (H99S) and 49 transcription factor mutant strains were grown in YPD liquid medium at 30 °C with shaking for 16 h, washed with PBS, and spotted onto Littman's (LIT) solid medium. Cells were further incubated for 2 days at 37 °C. Each graph indicates relative capsule size of transcription factor mutants that exhibited statistically significant changes in capsule production (±30% difference relative to wild type as cutoff). Two biologically independent experiments were performed and representative data are shown here. Each measurement was repeated for 20 cells per condition. Error bars indicate standard deviation. Statistical analysis was performed using one-way ANOVA with Bonferroni's multiple-comparison test. (*, $P < 0.05$; **, $P < 0.01$; ***, $P < 0.001$; ****, $P < 0.0001$). (B) WT (H99S) and 13 transcription factor mutant strains were grown in YPD liquid medium at 30 °C with shaking for 16 h, washed with PBS, and spotted onto FBS solid medium. Cells were further incubated for 2 days at 37 °C. Each graph indicates relative capsule size of transcription factor mutants that exhibited statistically significant changes in capsule production (±30% difference relative to wild type as cutoff). Two biologically independent experiments were performed and representative data are shown here. Each measurement was repeated for 20 cells per condition. Error bars indicate standard deviation. Statistical analysis was performed using one-way ANOVA with Bonferroni's multiple-comparison test (*, $P < 0.05$; **, $P < 0.01$; ***, $P < 0.001$; ****, $P < 0.0001$). (C) Expression level of *CAP* genes increases under capsule-inducing condition. The expression level of capsule biosynthesis genes was determined using qRT-PCR with cDNA from total RNA samples of WT (H99S) grown in basal YPD medium and three capsule-inducing media. *CAP10*, *CAP59*, *CAP60*, and *CAP64* expression levels were normalized by actin gene (*ACT1*) expression. Each strain grown in YPD medium (time zero sample) was re-suspended in Littman's (LIT) liquid medium, Dulbecco's Modified Eagle's (DME) liquid medium, and 10% fetal bovine serum (FBS) liquid medium and further incubated for 6 h. Three biological replicate samples with three technical replicates were analyzed using qRT-PCR. Error bars indicate standard deviation. Statistical analysis was performed using Student *t*-test (*, $P < 0.05$; **, $P < 0.01$; ***, $P < 0.001$; ****, $P < 0.0001$).

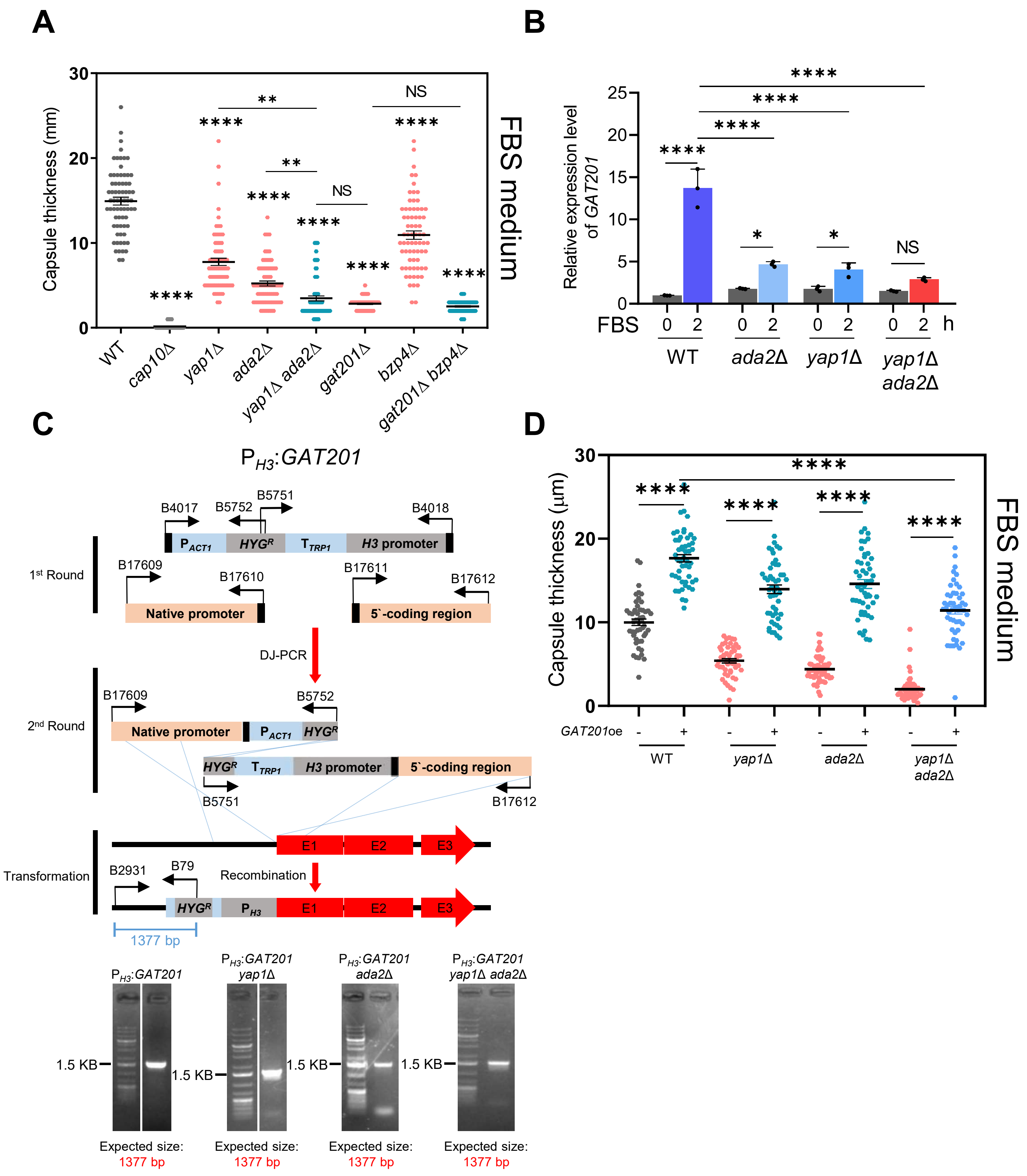

Figure S2. Yap1 and Ada2 co-regulate *GAT201* induction under capsule-inducing conditions. (A) WT (H99S), *cap10Δ* (YSB4081), *yap1Δ* (YSB815), *ada2Δ* (YSB2382), *yap1Δ ada2Δ* (YSB6054), *gat201Δ* (YSB3300), *bzp4Δ* (YSB1895), and *gat201Δ bzp4Δ* (YSB6052) strains were grown in YPD liquid medium at 30°C for 16 h, washed with PBS, and spotted onto 10% fetal bovine serum (FBS) solid medium. The cells were further incubated for 2 days at 37°C. Three biologically independent experiments were performed, and representative data are shown. Each measurement was repeated on 70 cells for each condition. Mean values were shown with error bars indicating standard deviation. Statistical analysis was performed using one-way ANOVA with Bonferroni's multiple comparison test (*, $P < 0.05$; **, $P < 0.01$; ***, $P < 0.001$; ****, $P < 0.0001$). (B) *GAT201* expression levels were determined using qRT-PCR with cDNA from total RNA samples of WT (H99S), *ada2Δ* (YSB2382), *yap1Δ* (YSB815), and *yap1Δ ada2Δ* (YSB6054) grown in basal YPD and 10% fetal bovine serum (FBS) medium. *GAT201* expression levels were normalized to actin gene (*ACT1*) expression. Each strain grown in YPD medium (time zero sample) was re-suspended in FBS liquid medium and further incubated for 2 h. Three biological replicate samples with three technical replicates were analyzed using qRT-PCR. Mean values were shown with error bars indicating standard deviation. Statistical analysis was performed using one-way ANOVA with Bonferroni's multiple-comparison test (*, $P < 0.05$; **, $P < 0.01$; ***, $P < 0.001$; ****, $P < 0.0001$). (C) Construction of the *GAT201* overexpression strains. The native promoter of *GAT201* was replaced with the constitutively active *H3* promoter linked to the hygromycin B resistance gene (see Methods and Materials). Positive transformants were confirmed by diagnostic PCR. All primer sets were listed in Table S1. The expected size of PCR-amplified DNA bands was indicated under each gel image. (D) *GAT201* overexpression strains generated in wild-type, *ada2Δ*, *yap1Δ*, or *yap1Δ ada2Δ* strain backgrounds were grown in YPD liquid medium at 30°C for 16 h, washed with PBS, and spotted onto FBS solid medium. The cells were further incubated for two days at 37°C. Each measurement was repeated on 50 cells for each condition Mean values were shown with error bars indicating standard deviation. Statistical analyses were performed using Student's *t*-test (* $P < 0.05$; ** $P < 0.01$; *** $P < 0.001$; **** $P < 0.0001$).

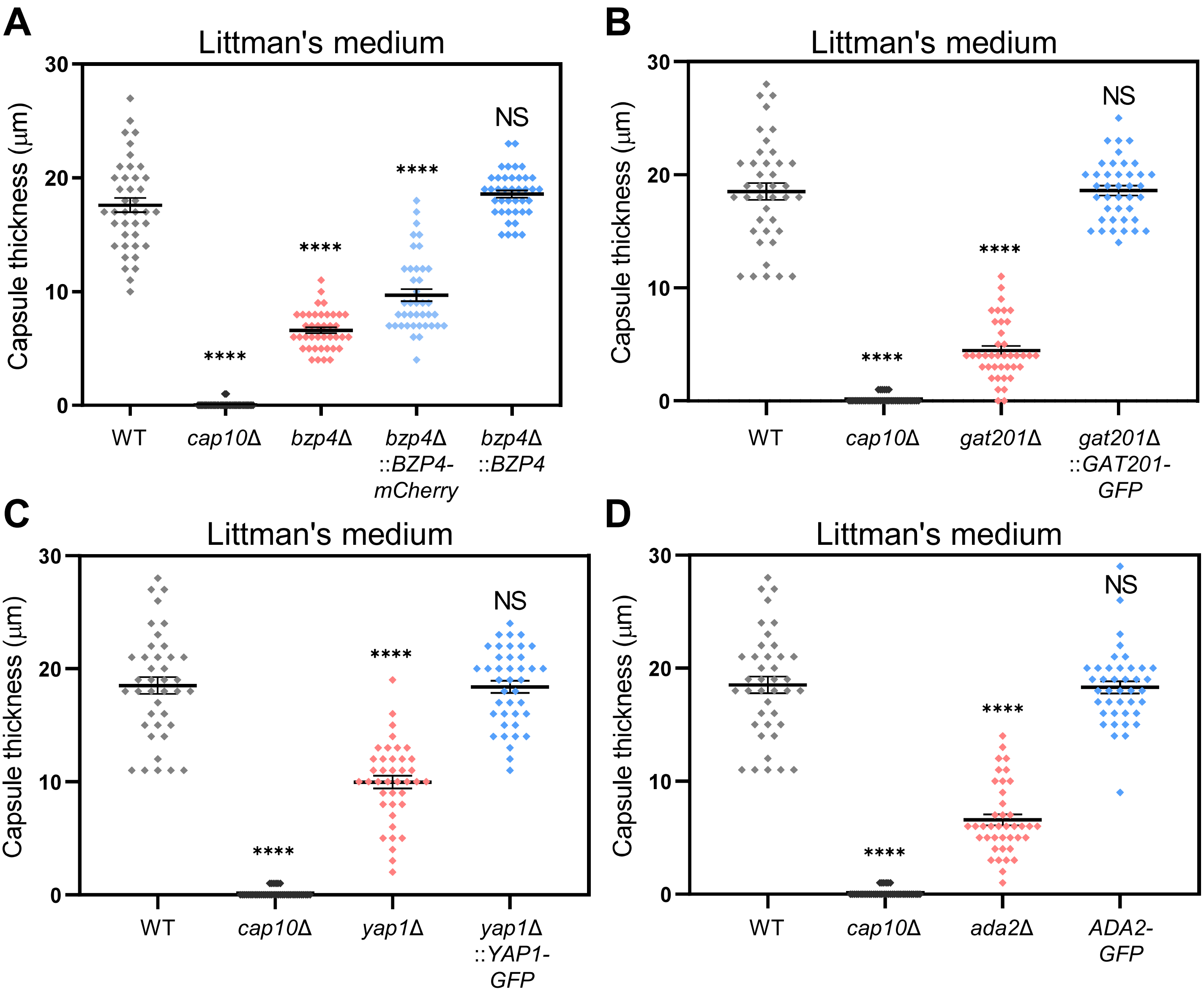

Figure S3. *BZP4*, *GAT201*, *YAP1*, and *ADA2* complemented strains showed similar capsule production level as the wild-type strain under capsule-inducing condition. WT (H99S), *cap10*Δ (YSB4081), (A) *bzp4*Δ (YSB1895), *bzp4*Δ::*BZP4-mCherry* (YSB5408), *bzp4*Δ::*BZP4* (YSB5499), (B) *gat201*Δ (YSB3300), *gat201*Δ::*GAT201-GFP* (YSB6745), (C) *yap1*Δ (YSB815), *yap1*Δ::*YAP1-GFP* (YSB2723), and (D) *ada2*Δ (YSB2382), *ADA2-GFP* (YSB3405) strains were grown in YPD liquid medium at 30 °C for 16 h, washed with PBS, and spotted onto a Littman's (LIT) solid medium. Cells were further incubated for 2 days at 37 °C. Two biologically independent experiments were performed and representative data are shown here. Each measurement was repeated for 40 cells per condition. Error bars indicate standard deviation. Statistical analysis was performed using one-way ANOVA with Bonferroni's multiple-comparison test (*, $P < 0.05$; **, $P < 0.01$; ***, $P < 0.001$; ****, $P < 0.0001$).

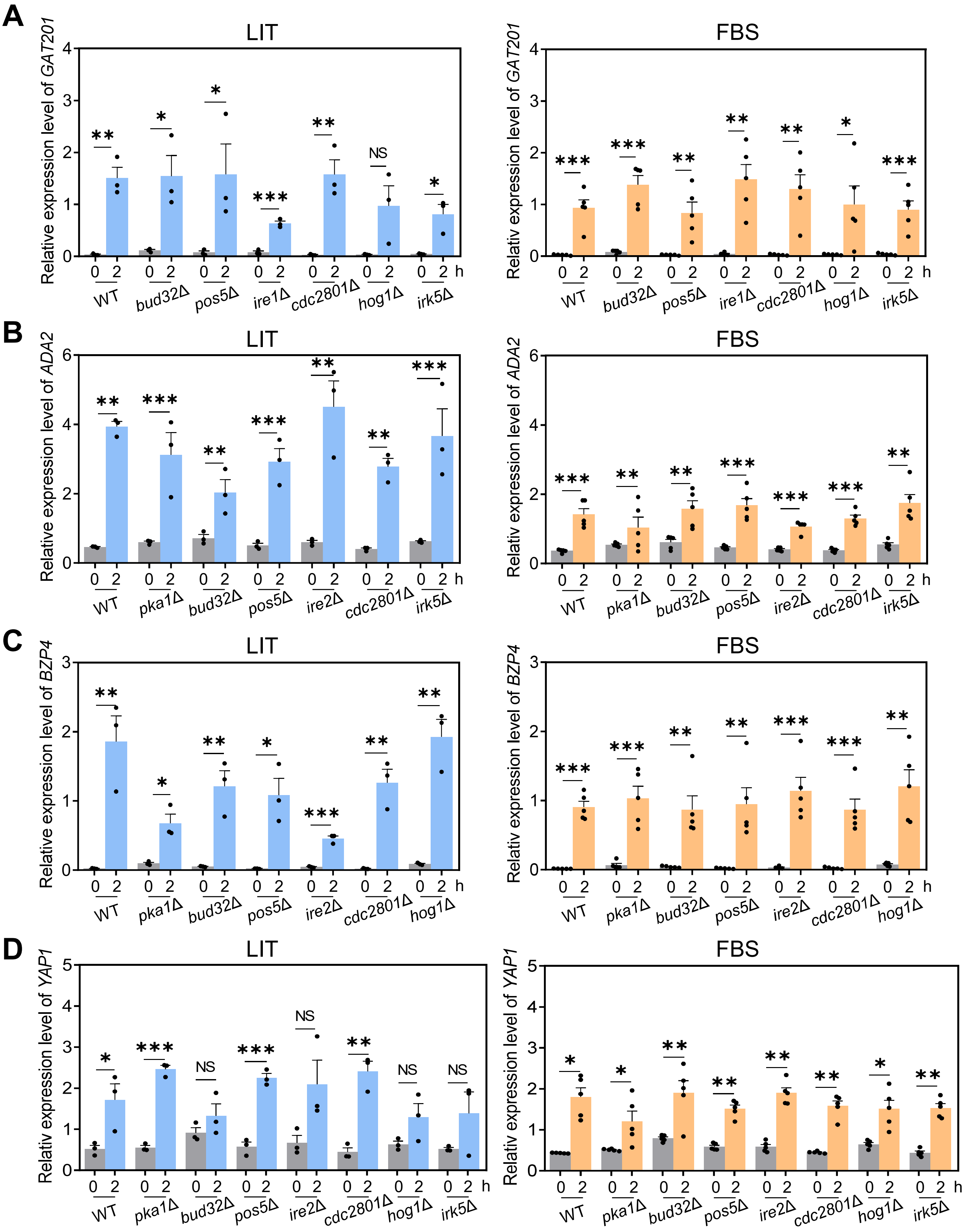

Figure S4. Multiple kinases regulate core capsule-regulating transcription factors under capsule-inducing condition. The expression level of *GAT201*, *YAP1*, *ADA2*, and *BZP4* was determined using qRT-PCR with cDNA from total RNA samples of WT (H99S), *pka1*Δ (YSB188), *bud32*Δ (YSB1969), *pos5*Δ (YSB3714), *ire1*Δ (YSB552), *cdc2801*Δ (YSB3699), *hog1*Δ (YSB64), and *irk5*Δ (YSB2952) grown in basal YPD and 10% fetal bovine serum (FBS) liquid medium. (A) *GAT201*, (B) *ADA2*, (C) *BZP4*, and (D) *YAP1* expression levels were normalized by actin gene (*ACT1*) expression. Each strain grown in YPD medium (time zero sample) was re-suspended in LIT or FBS liquid medium and further incubated for 2 h. Three to five biological replicate samples with three technical replicates were analyzed using qRT-PCR. Error bars indicate standard deviation. Statistical analysis was performed using Student *t*-test (*, $P < 0.05$; **, $P < 0.01$; ***, $P < 0.001$; ****, $P < 0.0001$).

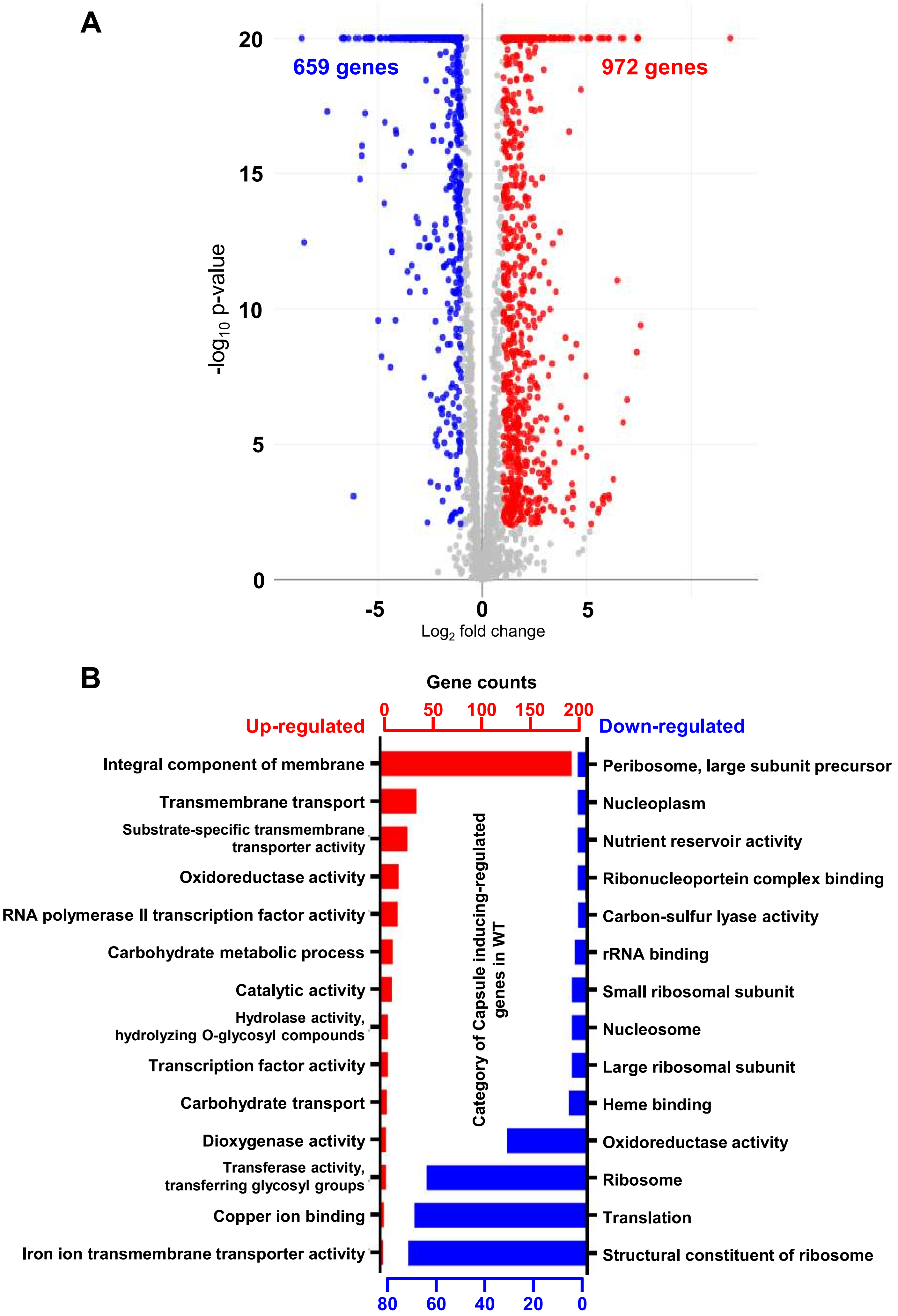

Figure S5. Capsule-inducing condition affects gene expression patterns of wild type strain. (A) Hierarchical clustering exhibits RNA-seq analysis of wild type (H99S) strain under basal and capsule-inducing condition (Littman's media). The cut-off range of the fold change was 2 with *P* value < 0.05 calculated using modified Fisher's exact test. Three independent biological experiments performed by each set. A volcano plot of the RNA data was constructed using DESeq2 and plotted using R. (B) DAVID analysis-based enrichment scores of gene ontology (GO) terms for genes upregulated (red) or downregulated (blue) in response tocapsule-inducing condition.

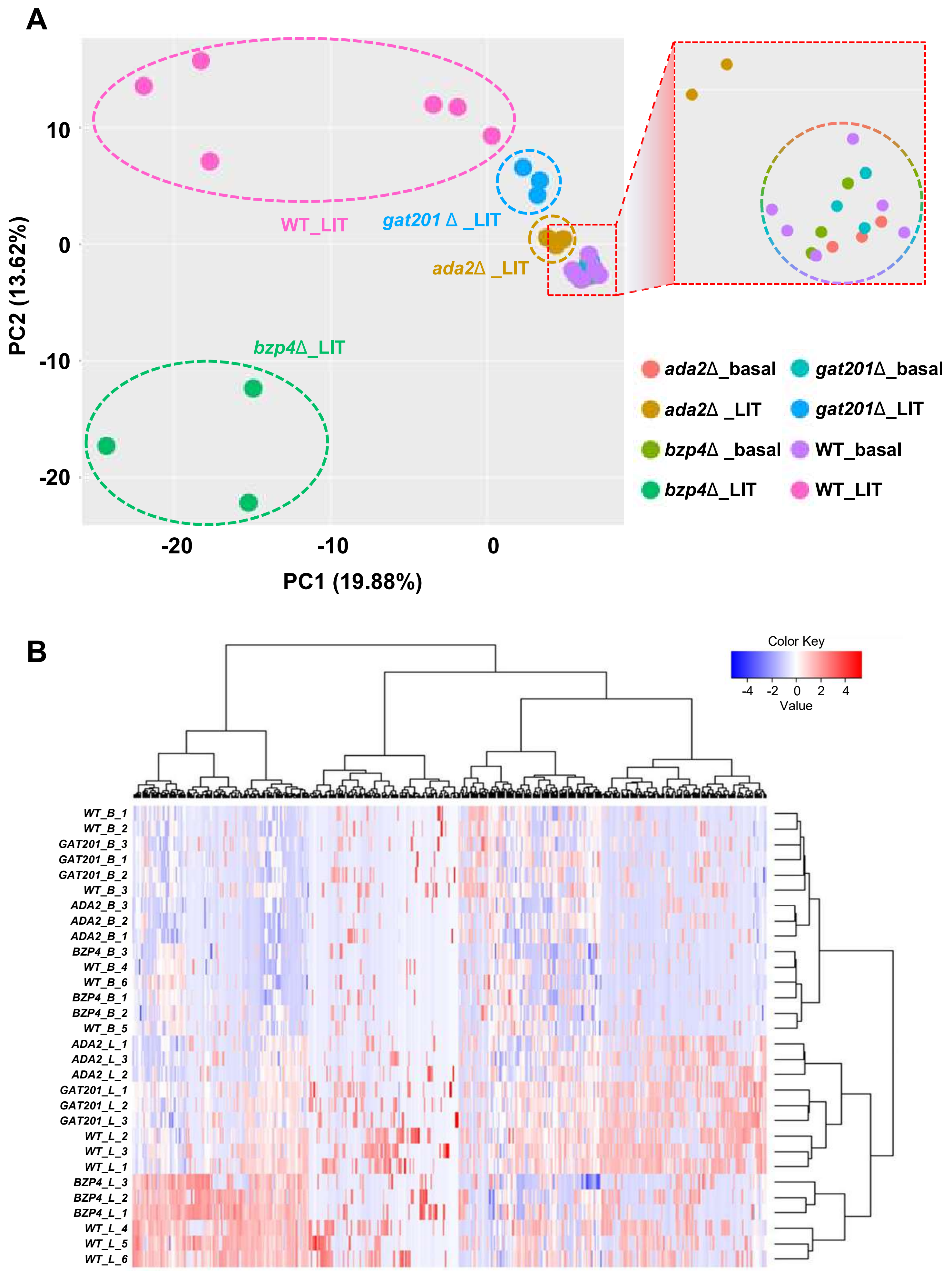

Figure S6. Capsule-inducing condition changes gene expression patterns of wild type strain. (A) Principal Component Analysis (PCA) of WT (H99S), *bzp4*Δ (YSB1895), *gat201*Δ (YSB3300), and *ada2*Δ (YSB2382) strains in basal and capsule-inducing conditions. (B) Hierarchical clustering and heatmap exhibit RNA-seq analysis of WT (H99S), *bzp4*Δ (YSB1895), *gat201*Δ (YSB3300), and *ada2*Δ (YSB2382) strains under basal and capsule-inducing conditions (Littman's media). A heatmap of RNA data that characterizes the role of the LIT treatment was constructed using DEBrowser.

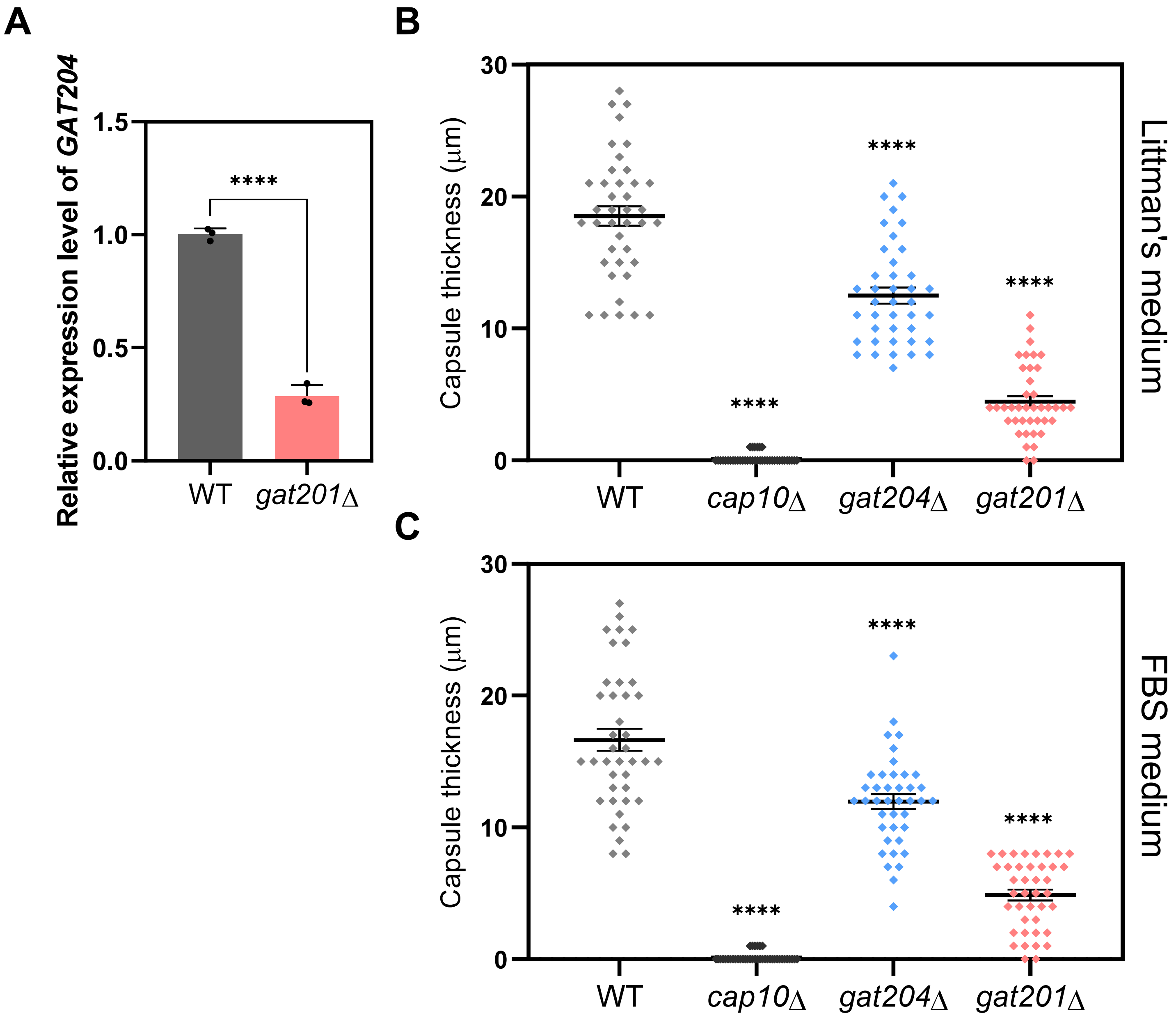

Figure S7. *GAT201* regulates the expression level of *GAT204*. (A) The expression level of *GAT204* was determined using qRT-PCR with cDNA from total RNA samples of WT (H99S) and *gat201*Δ (YSB3300) grown in basal YPD medium and LIT media. *GAT204* expression levels were normalized by actin gene (*ACT1*) expression. Each strain grown in YPD medium (time zero sample) was re-suspended in LIT liquid medium and further incubated for 2 h. Three biological replicate samples with three technical replicates were analyzed using qRT-PCR. Error bars indicate standard deviation. Statistical analysis was performed using Student *t*-test (*, $P < 0.05$; **, $P < 0.01$; ***, $P < 0.001$; ****, $P < 0.0001$). WT (H99S), *cap10*Δ (YSB4081), *gat204*Δ (YSB1311), and *gat201*Δ (YSB3300) were grown in YPD liquid medium at 30 °C shaking incubator for 16 h, washed with PBS, and spotted onto **(B)** LIT and **(C)** FBS solid medium. Cells were further incubated for 2 days at 37 °C. Each graph indicates relative capsule size of transcription factor mutants that exhibited statistically significant changes in capsule production (±30% difference relative to wild type as cutoff). Two biologically independent experiments were performed and representative data are shown here. Each measurement was repeated for 40 cells per condition. Error bars indicate standard deviation. Statistical analysis was performed using one-way ANOVA with Bonferroni's multiple-comparison test (*, $P < 0.05$; **, $P < 0.01$; ***, $P < 0.001$; ****, $P < 0.0001$).

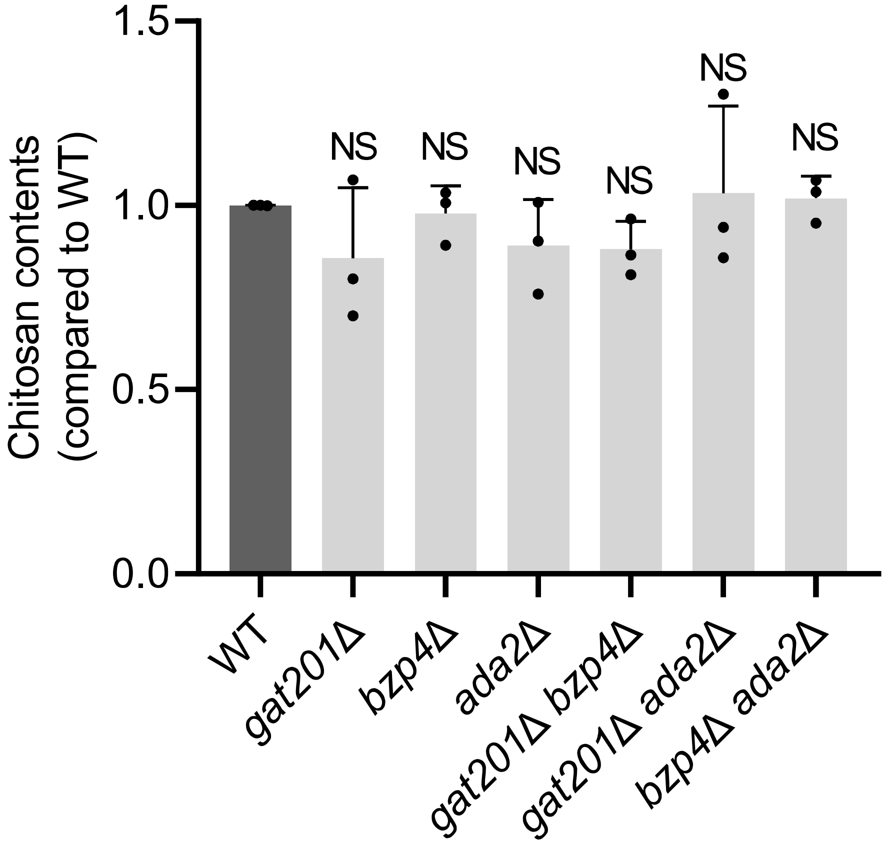

Figure S8. Measurement of the chitosan content on each mutant strain. Each strain was grown at 30°C in liquid medium for 2 days, collected by centrifugation, washed, and used in the 3-methyl-2-benzothiazolinone hydrazone hydrochloride (MBTH) assay for the quantitative measurement of chitosan. Three biological replicates are shown. Error bars indicate standard deviation. Statistical analysis was performed using one-way ANOVA with Bonferroni's multiple comparison test. (*, $P < 0.05$; **, $P < 0.01$; ***, $P < 0.001$; ****, $P < 0.0001$).
